# Potential Well in Poincaré Recurrence

**DOI:** 10.3390/e23030379

**Published:** 2021-03-23

**Authors:** Miguel Abadi, Vitor Amorim, Sandro Gallo

**Affiliations:** 1 Instituto de Matemática e Estatística, Universidade de São Paulo-USP, Rua do Matão, 1010 Butantã, São Paulo 05508-090, Brazil; 2 Instituto Federal de Educação, Ciência e Tecnologia de São Paulo-IFSP, Câmpus Araraquara, Rua Doutor Aldo Benedito Pierri, 250 Jardim Paulo Freire, Araraquara 14804-296, Brazil; vitoramorim@usp.br; 3 Departamento de Estatística, Universidade Federal de São Carlos-UFSCar, Rua dos Bem-te-vis, São Carlos 13565-905, Brazil

**Keywords:** potential well, poincaré recurrence times, exponential approximation, rare events, mixing processes

## Abstract

From a physical/dynamical system perspective, the potential well represents the proportional mass of points that escape the neighbourhood of a given point. In the last 20 years, several works have shown the importance of this quantity to obtain precise approximations for several recurrence time distributions in mixing stochastic processes and dynamical systems. Besides providing a review of the different scaling factors used in the literature in recurrence times, the present work contributes two new results: (1) For ϕ-mixing and ψ-mixing processes, we give a new exponential approximation for hitting and return times using the potential well as the scaling parameter. The error terms are explicit and sharp. (2) We analyse the uniform positivity of the potential well. Our results apply to processes on countable alphabets and do not assume a complete grammar.

## 1. Introduction

The close relation between the Extreme Value Theory (EVT) and the statistical properties of Poincaré recurrence has been recently quite well explored. The starting point is that the exceedances of a stochastic process to a sequence of barrier values $a_{n}>0,n\in N,$ can be considered as hittings to a sequence of nested sets. More precisely, if one defines the semi-infinite intervals:

$$A_{n}=\left(a_{n},\infty \right),$$

and considers a sequence of random variables $X_{1},X_{2},\ldots$, one has the equivalence:

$$\max\left\{X_{1},\ldots ,X_{t}\right\}>a_{n}\;\;\;\;\mathrm{if}\;\mathrm{and}\;\mathrm{only}\;\mathrm{if}\;\;\;\;\;T_{A_{n}}\leq t,$$

where for any measurable set *A*, $T_{A}$ denotes the smallest *k* such that $X_{k}\in A$. As the sequence of levels $a_{n}$ diverges, the sets $A_{n}$ are nested. This equivalence allows building a bridge between two historically independent theories: Extreme Value Theory (EVT) and Poincaré Recurrence Theory (PRT). While EVT focuses on the existence (and identification) of the limit of the distribution of the partial maxima and *k*-maxima, among others [1,2,3,4,5], the aim of recent works on PRT is to understand the statistical properties of the different notions of return times.

The present paper stands on the approach of PRT. Our interest is the statistics of visits of a random process $X_{t},t\in N$ to a given target measurable set. Asymptotic statistics are obtained by studying sequences of target sets $A_{n},n\geq 1$, usually of a measure shrinking to zero. In this context and for certain classes of processes, hitting and return times with respect to a given sequence of target sets converge to the exponential distribution, modelling the unpredictability of rare events. However, this rough affirmation is full of nuances that need to be established in very precise terms. It turns out that these details bring much information on the system.

For instance, for two observables having the same probability, the Ergodic theorem says that, macroscopically, their numbers of occurrences are about the same. However, these occurrences can appear scattered in a very different way along time. Under some strong mixing assumptions, it is a well-known fact of the literature that for nested sequences of observables with the same probability, the asymptotic observation of one of them can be distributed as a Poisson process while the other one will follow a compound Poisson process. Thus, the dichotomy Poisson/compound Poisson in the same system is determined also by the intrinsic properties of the target sets considered.

In the setting of the present paper, the target sets are finite strings of symbols (patterns). In this case, even if the process is a sequence of independent random variables, the successive occurrences of the string are not independent because the structure of the pattern itself enters the game, allowing or avoiding consecutive observations due to possible overlaps with itself. This leads to a dichotomy between aperiodic/periodic patterns that yields, in the limit of long patterns, the dichotomy Poisson/compound Poisson mentioned before. In passing, let us also mention that this dichotomy also exists in EVT where it is referred to as the phenomenon of clustering/non-clustering of maxima, and it has generated a great deal of research over the last two decades [3].

Let us now be more specific about what we are doing here. First, we operate in the context of discrete time stochastic processes with countable alphabet enjoying ϕ-mixing. Fix any point *x*, that is any right infinite sequence of symbols taken from the alphabet, and consider the nested sequence of neighbourhoods corresponding to the first *n* symbols of *x*, namely $A_{n}=\left(x_{0},\ldots ,x_{n-1}\right),n\geq 1$. The main theorem of the paper, Theorem 1, gives explicit and computable error terms for the approximation of the hitting time distribution $\mu (T_{A_{n}}>t)$ and return time distribution $\mu_{A_{n}}\left(T_{A_{n}}>t\right)$, by exponential distributions whose parameter is explicit and depends on $A_{n}$.

The first main advantage of Theorem 1 is that it uses the potential well as the scaling parameter. In other words, the potential well is the probability, conditioned on starting from $A_{n}$, that the pattern $A_{n}$ does not reappear at the first possible moment it could reappear. The use of this simple and well-defined quantity as the scaling parameter contrasts with previous works using parameters whose expressions are hardly explicit and even more hardly computable. Another advantage of Theorem 1 is that, unlike a whole body of literature obtaining almost sure results, our results hold for all *x*. This allows distinguishing different limiting distributions, as for example in the periodic/aperiodic dichotomy described above that almost sure results cannot detect. Finally, the error terms of our approximations are not in total variation distance, but in the stronger point-wise form with respect to the time scale.

The closest result of the literature, but restricted to return times, is the main theorem of [6]. Our Theorem 1, besides considering also the case of hitting times, also extends the class of processes to which the approximations apply. First, we do not assume the finiteness of the alphabet, allowing countably infinite alphabets. Second, our processes are not assumed to have a complete grammar, as was the case of [6]. In ergodic theory terminology, this means that we do not restrict to the full shift. Yet another contribution here is that we specify a tighter and simpler error term under the stronger assumption of ψ-mixing in both hitting and return time approximations.

Last but not least, Theorem 1 corrects the exponential approximation obtained by [6] for return times. Indeed, the error term of their Theorem 4.1 contains a mistake for small *t*’s.

The other main novelty of the present work is Theorem 2, stating that (1) the potential well almost surely converges to one as the size of the patter diverges under ϕ-mixing and (2) the potential well is uniformly bounded away from zero when we have ψ-mixing or ϕ-mixing with summable function ϕ(n). Naturally, as a conditional probability, we know that the potential well belongs to the interval [0,1] for any n≥1 and any pattern $A_{n}$. However, it was proven that the potential well could be arbitrarily close to zero for β-mixing processes, a slightly weaker mixing assumption than ϕ-mixing. Indeed, it was shown in [7] that for the binary renewal process, with specific choices of transition probabilities and target sets $A_{n},n\geq 1$, the potential well of $A_{n}$ vanishes as *n* diverges. Note that the border is thin between this β-mixing example and our Theorem 2 holding for ψ-mixing and ϕ-mixing with summable ϕ (see the review of [8] on the distinct mixing assumptions). We conjecture that the assumption of the summability of the ϕ rates can be dropped.

A fundamental quantity is the shortest possible return of the pattern $A_{n}$, denoted $\tau (A_{n})$, which is in particular used to define the potential well. It is known [9,10] that under the assumptions of specification and positive entropy, $\tau (A_{n})/n$ converges almost surely to one. Since we do not assume a complete grammar, $\tau (A_{n})$ is not bounded above by *n*, and it could be strictly larger than *n* in general. Here, we prove an important technical result (Lemma 2) giving explicit uniform upper bounds, which hold for sufficiently large *n* and under either ϕ- or ψ-mixing.

To conclude about the importance of the present work as a whole, let us mention that our results are fundamental for the study of further recurrence quantities, such as the return time function [11,12] and the waiting time function [13,14], establishing a link with information theory. These random variables are known to satisfy a counterpart of the famous Shannon–McMillan–Breiman theorem (asymptotic equipartition property). In order to study the fluctuations of these limit theorems, for instance a large deviation principle, we need to control the return/hitting time exponential approximations for any point and any t>0. This was particularly clear in [15,16], studying the fluctuations of the waiting time and return time, respectively. It is also interesting to notice that it was [17] who first pointed out the importance of seeking exponential approximations for any point *x*, and it was precisely to study the small and large fluctuations of the return time function.

The paper is organized as follows. We describe in Section 2 the setting of the paper in the context of PRT, defining carefully the types of exponential approximations we are interested in and explaining, including through an extensive bibliography, the role of the potential well as the scaling parameter. Section 3 contains the main results, and Section 4 is dedicated to their proofs.

## 2. Poincaré Recurrence Theory for Mixing Processes

### 2.1. The Framework of Mixing Processes

Consider a countable set A that we call the alphabet. By N, we denote the set of nonnegative integers and, by $X:=A^{N}$, the set of right infinite sequences $x=(x_{0},x_{1},\ldots )$ of symbols taken from A. Given a point x∈X and for any finite set I⊂N, the cylinder sets with the base in *I* is defined as the set $A_{I}\left(x\right):=\left\{y\in X:y_{i}=x_{i},i\in I\right\}$. In the particular case where I={0…,n−1}, we write $A_{n}\left(x\right)$ and sometimes abuse notation, writing $x_{0}^{n-1}$. We endow X with the σ-algebra F generated by the class of cylinder sets $\left\{A_{I}:I\subset N,|I|<\infty \right\}$. Furthermore, $F_{I}$ denotes the σ-algebra generated by $A_{I}\left(x\right),x\in X$. For the special case in which I={i,…,j}, 0≤i≤j≤∞, we use the notation $F_{i}^{j}$. We use the shorthand notation $a_{i}^{j}:=\left(a_{i},a_{i+1},\ldots ,a_{j}\right)$, 0≤i≤j<∞ for finite strings of consecutive symbols of A. When necessary, $A_{n}\left(x\right)$ will be naturally identified with the sequence $x_{0}^{n-1}$.

The shift operator σ:X→X shifts the point $x=(x_{0},x_{1},x_{2},\ldots )$ to the left by one coordinate, ${\left(\sigma x\right)}_{i}=x_{i+1}$, i≥0.

We consider a shift invariant (or stationary) probability measure μ on (X,F). For any A∈F of positive measure, $\mu_{A}\left(\cdot \right):=\frac{\mu (\{x\in \cdot \cap A\})}{\mu (A)}$ is the conditional measure μ restricted to *A*.

Our results are stated under two mixing conditions that we now define. For all n≥1, define:

$$\begin{array}{cc} \phi (n) & :=\underset{i\in N,A\in F_{0}^{i},B\in F_{i+n}^{\infty }}{\sup}\left|\frac{\mu (A\cap B)}{\mu (A)}-\mu \left(B\right)\right|,\\ \psi (n) & :=\underset{i\in N,A\in F_{0}^{i},B\in F_{i+n}^{\infty }}{\sup}\left|\frac{\mu (A\cap B)}{\mu (A)\mu (B)}-1\right|. \end{array}$$

Note that ψ(n) and ϕ(n) are nonincreasing sequences, since $F_{0}^{i}\subset F_{0}^{i+1}$ for every i≥0.

**Definition** **1.** *We say that the measure μ on (X,F) is ϕ-mixing (resp. ψ-mixing) if ϕ(n) (resp. ψ(n)) goes to zero as n diverges. We say that μ is “summable ϕ-mixing” if it is ϕ mixing with $\sum_{n}\phi \left(n\right)<\infty$.*

We refer to [8] for an exhaustive review of mixing properties and examples.

### 2.2. Recurrence Times and Exponential Approximations

The hitting time of a point *y* to a set A∈F is defined by:

$$\begin{array}{cc} T_{A}\left(y\right) & =\inf\{k\geq 1:\sigma^{k}\left(y\right)\in A\}. \end{array}$$

For sets *A* of small measure (rare events) and under mixing conditions such as the ones introduced in the preceding subsection, it is expected that $\mu (T_{A}>t)$ is approximately exponentially distributed. This is what we call hitting time exponential approximation. Similarly, when we refer to return time, we mean that we study the approximation of $\mu_{A}\left(T_{A}>t\right)$, that is the measure of the same event, conditioned on the points starting in *A*.

In this paper, we are interested in the case where we fix any point *x* and consider $A_{n}\left(x\right)$ as the target set. When *n* diverges, the measure of $A_{n}\left(x\right)$ vanishes, leading to rare events. The scaling parameter of the exponential approximation depends on the point *x*.

The two main types of approximations that appeared in the literature when approximating the hitting/return time distributions around any point *x* of the phase space are a total variation distance type and a pointwise type.

- Type 1: Total variation distance. For any x∈X,
  - Hitting times:
    $$\underset{t>0}{\sup}\left|\mu \left(T_{A}>t\right)-e^{-\mu (A)\theta (A)t}\right|\leq \epsilon \left(A\right),$$
  - Return times:
    $$\underset{t>0}{\sup}\left|\mu_{A}\left(T_{A}>t\right)-\bar{\theta }\left(A\right)e^{-\mu (A)\theta (A)t}\right|\leq \epsilon \left(A\right).$$
- Type 2: Pointwise. For any x∈X and any t>0,
  - Hitting times:
    $$\left|\mu \left(T_{A}>t\right)-e^{-\mu (A)\theta (A)t}\right|\leq \epsilon \left(A,t\right),$$
  - Return times:
    $$\left|\mu_{A}\left(T_{A}>t\right)-\bar{\theta }\left(A\right)e^{-\mu (A)\theta (A)t}\right|\leq \epsilon \left(A,t\right).$$

Note that in the return time approximation, the parameters θ and $\bar{\theta }$ need not be equal. However, such approximation leads to:

$$E_{A}\left(T_{A}\right)\approx \bar{\theta }\left(A\right)\frac{1}{\mu (A)\theta (A)}.$$

In view of Kac lemma, which, we recall, states that $E_{A}\left(T_{A}\right)=\frac{1}{\mu (A)}$, the last display suggests that θ and $\bar{\theta }$ must be close.

### 2.3. Potential Well: Definition and Genealogy in PRT

As already explained, the potential well will be used as the scaling parameter in the exponential approximations of Types 1 and 2 defined above. In order to define it, we need first to define the shortest possible return of a set A∈F (to itself):

$$\tau \left(A\right):=\underset{y\in A}{\inf}\left\{T_{A}\left(y\right):\mu_{A}\left(\sigma^{-T_{A}\left(y\right)}\left(A\right)\right)>0\right\},$$

or, equivalently:

$$\tau \left(A\right):=\inf\left\{k\geq 1:\mu_{A}\left(\sigma^{-k}\left(A\right)\right)>0\right\}.$$

In the case where $A=A_{n}\left(x\right)$, we define $\tau_{n}\left(x\right)=\tau \left(A_{n}\left(x\right)\right)$. The $\tau_{n}:X\to N$ constitutes a sequence of simple functions. Notice that the above alternative definitions of τ(A) involve the measure $\mu_{A}$, while the traditional definition is completely topological. This is to account for the case where the measure does not have a complete grammar (We say that μ has a complete grammar if $\mu (A_{n}\left(x\right))>0$ for any x∈X and n≥1).

The first possible return time $\tau_{n}\left(x\right)$ is an object of independent interest, which was studied under several perspectives in the literature. Let us mention that its asymptotic concentration was proven by [9,10], large deviations in [18,19,20], and fluctuations in [21,22].

Obviously, by definition, $\mu_{A}\left(T_{A}\geq \tau \left(A\right)\right)=1$. If for a point x∈A, we have $T_{A}\left(x\right)>\tau \left(A\right)$, we say that *x* escapes from *A*. The potential well of order *n* at *x* is precisely the proportional measure of points of *A* that escape from *A*:

$$\rho \left(A\right):=\mu_{A}\left(T_{A}>\tau \left(A\right)\right).$$

Since we are interested in the case where $A=A_{n}\left(x\right)$, we use the alternative notation $\rho (x_{0}^{n-1})$ instead of $\rho (A_{n}\left(x\right))$. Besides being explicitly computable in many situations, the potential well is physically meaningful and, as the scaling parameter, provides precise exponential approximations for recurrence times under suitable mixing assumptions.

We give below a small genealogy of scaling parameters that appeared in the literature that consider results holding for all points to get approximations for hitting/return times.

- As far as we know, the first paper to prove exponential approximations for hitting time statistics for all points is due to Aldous and Brown [23]. They obtained Type 1 approximations in the case of reversible Markov chains. The parameter used there was just the inverse of the expectation, which is mandatory to use when the approximating law is the exponential distribution. However, this does not bring information about the value of the expectation.
- Galves and Schmitt [24] obtained Type 1 approximations for hitting times in ψ-mixing processes. The major breakthrough there was that the authors provided an explicit formula for the parameter (denoted by λ(A)). This quantity could be viewed as the grandfather of ρ. Nonetheless, its explicit significance was not evident.
- References [25,26] gave exponential approximations (Type 1 and Type 2, respectively) of the distribution of hitting time around any point using a scaling parameter. In [26], however, only its existence and necessity were proven, the calculation of λ being intractable in general. The main problem is that λ(A) depends on the recurrence property of the cylinder *A* up to large time scales (usually of the order of $\mu {\left(A\right)}^{-1}$).
- In order to circumvent this issue, Reference [25] also provided, in the context of approximations of Type 1, another scaling parameter, easier to compute, but with a slightly larger error term as a price to pay. It is defined as follows:
  $$\zeta_{s}\left(x_{0}^{n-1}\right):=\mu_{x_{0}^{n-1}}\left(T_{x_{0}^{n-1}}>n/s\right).$$
  This quantity depends on, at most, the 2n first coordinates of the process. $\zeta_{s}\left(x_{0}^{n-1}\right)$ can be seen as the father of the potential well. Both works [25,26] led with processes enjoying ψ-mixing or summable ϕ-mixing.
- The use of the potential well ρ as the scaling parameter was firstly proposed by Abadi in [27], still in the context of an approximation of Type 1 for hitting and return times. More specifically, it is proven that, for exponentially α-mixing processes, λ and ζ (grandpa and father of ρ) can be well approximated by ρ.
- The first paper to really directly use ρ as the scaling parameter was [6], in which a Type 2 approximation for return times was obtained, with $\bar{\theta }=\theta =\rho$. The process is assumed to be ϕ-mixing.
- Focusing on proving exponential approximations for hitting and return times under the largest possible class of systems, and still for all points, Abadi and Saussol [28] returned to the approach of Galves and Schmitt. Their results hold under the α-mixing condition, which is the weakest hypothesis used up to date, but the scaling parameter is not explicit.
- Focussing on the specific class of binary renewal processes, Reference [7] proved a Type 1 approximation for hitting and return times using the potential well ρ. One interesting aspect concerning this work lies in the fact that the renewal process is β-mixing (weaker than the ϕ-mixing assumed by [6]). Moreover, the authors managed to use the renewal property to compute the limit of $\rho (A_{n}\left(x\right))$ for any point *x*. In other words, the approximating asymptotic law for hitting and return times was explicitly computed as a function of the parameters of the process. This result shows the usefulness of the potential well, an “easy to compute” scaling parameter.

## 3. Main Results

Theorem 1 below presents Type 2 approximations for hitting and return time under ϕ- and ψ-mixing conditions with the potential well as the scaling parameter and an explicit error term.

Before we can state this result, we first need to define the second order periodicity of string $A_{n}\left(x\right)$, which plays a crucial role for the size of the error term.

### 3.1. Second Order Periodicity

The short returns that we will define here are precisely those that are difficult to treat as (almost) independent. They not only depend on the correlation decay of the system, but also on the particular properties of the string itself. Technically, for an *n*-cylinder, short means returning in up to the order *n* steps.

Consider the cylinder *A*, and suppose τ(A)=k. Write n=qk+r, where q∈N and 0≤r<k, and note that the cylinder overlaps itself in all multiples of *k* smaller than *n*. The set P(A):={mk:1≤m≤q} s the indexes of possible returns at multiples of τ(A), but returns can also occur at other time indexes after that. Let:

$$R\left(A\right)=\{j\in \left\{qk+1,\ldots ,qk+r-1\right\}:\;\mu_{A}\left(\sigma^{-j}\left(A\right)\right)>0\}.$$

A point y∈A could only return to *A* before *n* at time indexes in P(A)∪R(A), but there is a crucial difference between them. A point that escapes from *A* cannot return in P(A), but it could return in R(A). Namely,

$$\mu_{A}\left(\sigma^{-\tau (A)}\left(A^{c}\right)\cap \sigma^{-j}\left(A\right)\right)=0,\;\;j\in P\left(A\right),$$

while:

$$\mu_{A}\left(\sigma^{-\tau (A)}\left(A^{c}\right)\cap \sigma^{-j}\left(A\right)\right)>0,\;\;j\in R\left(A\right).$$

We set $n_{A}$ as the first possible return to *A*, among those points x∈A that escape *A* at τ(A):

$$n_{A}=\min\left\{j:\mu_{A}\left(\sigma^{-\tau (A)}\left(A^{c}\right)\cap \sigma^{-j}\left(A\right)\right)>0\right\}.$$

We refer to [6] for an example that illustrates these ideas under a complete grammar and a finite alphabet. In the general case, notice that $n_{A}$ could be strictly lager than *n*, if one is not considering the full shift. For a complete example, consider the house of cards Markov chain. It has transition matrix *Q* on N with entries $Q\left(i,0\right)=q_{i}=1-Q\left(i,i+1\right)$ where $q_{i},i\geq 0$ is a sequence of real numbers taking values in the interval (0,1). Therefore, it is defined on an infinite alphabet and does not have a complete grammar, since several transitions are forbidden due to the sparse nature of *Q*. Consider the strings A=00010001000 and B=(n+1)…2n (for some n≥1) generated by this Markov chain in the stationary measure. Then, we see that τ(A)=4, R(A)={9,10}, and $n_{A}=9$, while, on the other hand, τ(B)=2n+1>n, R(B)=∅, and $n_{B}=3n+2$.

### 3.2. Type 2 Approximations Scaled by the Potential Well

For any finite string *A*, let us denote by $A^{\left(k\right)}$ the suffix of *A* of size *k*. That is, if $A=x_{0}^{n-1}$, then $A^{\left(k\right)}=x_{n-k}^{n-1}$. When *A* is an *n*-cylinder we use the convention $\mu \left(A^{\left(j\right)}\right)=\mu \left(A^{\left(n\right)}\right)=\mu \left(A\right)$ for j≥n.

By definition, ϕ(g) is finite for all g≥1. This is not the case for ψ(g). Thus, for ψ-mixing measures, we define:

(1) $$g_{0}=g_{0}\left(\psi \right):=\inf\left\{g\geq 1:\psi \left(g\right)<\infty \right\}-1.$$

Now, for the error term, define:

(2) $$\begin{array}{cc} \; & \left(a\right)\;\;\epsilon_{\psi }\left(A\right):=n\mu \left(A^{\left(n_{A}-g_{0}\right)}\right)+\psi \left(n\right), \end{array}$$

(3) $$\begin{array}{cc} \; & \left(b\right)\;\;\epsilon_{\phi }\left(A\right):=\underset{1\leq w\leq n^{*}}{\inf}\left\{\left(n+\tau \left(A\right)\right)\mu \left(A^{\left(w\right)}\right)+\phi \left(\min\left\{n_{A}-w+1,n\right\}\right)\right\}. \end{array}$$

where $n^{*}:=\min\left\{n,n_{A}\right\}$.

Note that cylinders *A* of size *n* verify that $n_{A}\geq n/2$, then $\epsilon_{\psi }$ is well defined for all $n>2g_{0}$.

We use ϵ to denote either $\epsilon_{\psi }$ or $\epsilon_{\phi }$ when the argument/statement is general.

**Theorem** **1.** *Consider a stationary measure μ on (X,F) enjoying either ϕ-mixing with $\sup_{A\in A^{n}}\mu \left(A\right)\tau \left(A\right)\overset{n}{⟶}0$, or simply ψ-mixing. There exist five positive constants $C_{i}$, i=1,…,5, and $n_{0}\in N$ such that for all $n\geq n_{0}$ and all $A\in A^{n}$, the following inequalities hold.*

- *For all*t≥0:

$$\;\left|\mu \left(T_{A}>t\right)-e^{-\rho (A)\mu (A)t}\right|\leq \left\{\begin{array}{cc} C_{1}\left(\tau (A)\mu (A)+t\mu (A)\epsilon (A)\right) & t\leq {\left[2\mu \left(A\right)\right]}^{-1}\\ C_{2}\;\mu \left(A\right)t\epsilon \left(A\right)e^{-\mu \left(A\right)t\left(\rho \left(A\right)-C_{3}\epsilon \left(A\right)\right)} & t>{\left[2\mu \left(A\right)\right]}^{-1}. \end{array}\right.$$

*For all*t≥τ(A):

$$\left|\mu_{A}\left(T_{A}>t\right)-\rho \left(A\right)e^{-\rho (A)\mu (A)(t-\tau (A))}\right|\;$$

$$\;\leq \left\{\begin{array}{cc} C_{4}\;\epsilon \left(A\right) & t\leq {\left[2\mu \left(A\right)\right]}^{-1}\\ C_{5}\;\mu \left(A\right)t\epsilon \left(A\right)e^{-\mu \left(A\right)t\left(\rho \left(A\right)-C_{3}\epsilon \left(A\right)\right)} & t>{\left[2\mu \left(A\right)\right]}^{-1}. \end{array}\right.$$

Theorem 1 (and its proof) were definitely inspired by [6] and their Theorem 4.1. However, let us first observe that our result provides the first statement of the literature for Type 2 hitting time approximations with the potential well as the scaling parameter. Moreover, contrary to [6], we do not assume a complete grammar nor a finite alphabet.

Let us make some further important observations concerning this theorem.

**Remark** **1.** *Under ϕ-mixing, the assumption $\sup\mu \left(A\right)\tau \left(A\right)\overset{n}{⟶}0$ can be dropped under certain circumstances. For instance, if the measure has a complete grammar, we have τ(A)≤n, and the assumption is granted using Lemma 1. Another way is to assume that μ is* summable *ϕ-mixing, as mentioned after Lemma 2 in Section 4.*

**Remark** **2.** *According to Lemma 1, if μ is ϕ-mixing (and a fortiori, ψ-mixing), there exist constants C and c such that $\mu \left(A\right)\leq Ce^{-cn}$ for all n≥1 and $A\in A^{n}$. On the other hand, since $n_{A}\geq n/2$, we get $\mu \left(A^{\left(n_{A}-g_{0}\right)}\right)\leq Ce^{-c(n/2-g_{0})}$ for all $n>2g_{0}$. Therefore, $\epsilon_{\psi }\left(A\right)\overset{n}{⟶}0$ uniformly. Further, if τ(A)≤2n, it is enough to take w=⌈n/4⌉ to obtain $\phi \left(\min\left\{n_{A}-w+1,n\right\}\right)\leq \phi \left(\left\lfloor n/4\right\rfloor \right)$ and $\left(n+\tau \left(A\right)\right)\mu \left(A^{\left(w\right)}\right)\leq 3Cne^{-cn/4}$, which ensures $\epsilon_{\phi }\left(A\right)\overset{n}{⟶}0$ uniformly. This is the case, for instance, if one has a complete grammar. On the other hand, notice that $\tau \left(A\right)<n_{A}$. Hence, if τ(A)>2n, we take w=n and get $\phi \left(\min\left\{n_{A}-w+1,n\right\}\right)=\phi \left(n\right)$. Therefore, since $\tau \left(A\right)\mu \left(A\right)\overset{n}{⟶}0$, we also have in this case $\epsilon_{\phi }\overset{n}{⟶}0$.*

**Remark** **3.** *Naturally, the statements under ψ-mixing are less general, but have smaller error terms. The error term is the same for $t>{\left[2\mu \left(A\right)\right]}^{-1}$ for both hitting and return times’ approximations. The difference is for small t’s, due to the correlation arising from the conditional measure.*

**Remark** **4.** *For application purposes involving data, it is essential to control all the constants involved in the statements. These constants can be accessed from the proof presented in Section 4.2, where we also make explicit the integer $n_{0}$ from which Theorem 1 holds (see (23)). If μ is ψ-mixing, we define $M:=\psi \left(g_{0}+1\right)+1$. In this case, $C_{1}=8M+9$, $C_{2}=194M+206$, $C_{3}=66M+89$, $C_{4}=12M+15$, and $C_{5}=197M+220$. On the other hand, for the ϕ-mixing case, we have $C_{1}=9$, $C_{2}=143$, $C_{3}=61$, $C_{4}=14$, and $C_{5}=170$.*

**Remark** **5.** *We show the sharpness of the error term in the return time approximation given by Theorem 1 with a simple example. Consider an i.i.d. process ${\left(X_{m}\right)}_{m\in N}$ with alphabet A. Take b∈A such that μ(b)=p and $x=\left(b,b,\ldots \right)\in X=A^{N}$. Thus, $A_{n}\left(x\right)=x_{0}^{n-1}=\left(b,b,\ldots ,b\right)$. Direct calculations give:*

- $\mu \left(A_{n}\right)=p^{n}$
- $\tau (A_{n})=1$
- $\rho \left(A_{n}\right)=1-\mu_{A_{n}}\left(T_{A_{n}}=\tau \left(A_{n}\right)\right)=1-\mu_{A_{n}}\left(X_{n}=b\right)=1-p$
- $n_{A_{n}}=n$
- $\mu_{A_{n}}\left(T_{A_{n}}>n-1\right)=\rho \left(A_{n}\right)=1-p$.

*An i.i.d. process is trivially ψ-mixing with function ψ identically zero. Thus, Theorem 1 states that the error for small *t*’s is $\epsilon_{\psi }\left(A_{n}\right)=np^{n}$. On the other hand, by direct substitution using the above facts, we have for each n≥2*:

$$\begin{array}{cc} \left|\mu_{A_{n}}\left(T_{A_{n}}>n-1\right)-\rho \left(A_{n}\right)e^{-\rho \left(A_{n}\right)\mu \left(A_{n}\right)\left(\left(n-1\right)-\tau \left(A_{n}\right)\right)}\right| & =\left|\left(1-p\right)-\left(1-p\right)e^{-\left(1-p\right)p^{n}\left(\left(n-1\right)-1\right)}\right|\\ \; & =\left(1-p\right)\left(1-e^{-\left(1-p\right)p^{n}\left(n-2\right)}\right) \end{array}$$

*which implies that the exact error in the approximation for the return time at n−1 is of order $p^{n}n$, just as stated by Theorem 1.*

**Remark** **6.** *The reader may notice a difference between Theorem 1 and Theorem 4.1 of [6] concerning the error term for small t’s for return time approximation. Indeed, their statement is incorrect as shown by the preceding example. We recall that the error term for small t’s plays a fundamental role when studying the return time spectrum, as was done by [15]. Theorem 1, besides correcting [6], is also fundamental to correct [15], which was based on the exponential approximations given by [6].*

**Remark** **7.** *As an example where Theorem 1 can be applied while Theorem 4.1 of [6] cannot, let us consider the house of cards Markov chain defined in the previous subsection. We refer to [7] where it was explained that, if $q_{i}\geq \epsilon ,i\geq 1$ for some ϵ>0, there exists a stationary process ${\left(X_{m}\right)}_{m\in N}$ with matrix Q, and it is ϕ-mixing with exponentially decaying ϕ(n). Therefore, according to Remark 1, it fits into the conditions of Theorem 1. However, since it is defined on an infinite alphabet and does not have a complete grammar, such processes are not covered by [6].*

### 3.3. Uniform Positivity of the Potential Well

Theorem 1 says that the potential well can be used as the scaling parameter to obtain approximations for recurrence times around any point. We now ask about the possible values of this scaling parameter in its range [0,1].

Abadi and Saussol [29], in the more general case known up to now, proved that for α-mixing processes with at least polynomially decaying α rates, the distribution of the hitting and return time converges, almost surely, to an exponential with parameter of one. We refer to [8] for the precise definition of α-mixing, but the only important point for us it to know that summable ϕ-mixing implies α-mixing with at least polynomially decaying α rates. This fact, combined with Theorem 1, proves, indirectly, that for summable ϕ-mixing processes, the potential well converges almost surely to one, since both theorems must agree on the limiting distribution under these conditions. Theorem 2 Item (a) below states that the same holds for ϕ-mixing without any assumption on the rate.

On the other hand, for the renewal processes, with a certain tail distribution for the inter-arrival times, Abadi, Cardeño, and Gallo [7] proved that for the point x=(00000…), the sequence of potential wells $\rho (x_{0}^{n-1})$ converges to zero. In this case, the scaling parameter has a predominant role, indicating the drastic change of scale of the occurrence of events. For instance, in this case, the mean hitting time is much larger than the mean return time:

$$E\left(T_{x_{0}^{n-1}}\right)\approx \frac{1}{\rho \left(x_{0}^{n-1}\right)\mu \left(x_{0}^{n-1}\right)}\gg \frac{1}{\mu (x_{0}^{n-1})}=E_{x_{0}^{n-1}}\left(T_{x_{0}^{n-1}}\right).$$

Such renewal processes are β-mixing (see [8] for the definition). Theorem 2 Item (b) below states that this cannot happen for ψ-mixing processes or summable ϕ-mixing processes.

**Theorem** **2.** *Let μ be a stationary ϕ-mixing measure. Then:*

*(a)*  *$\rho \left(x_{0}^{n-1}\right)\overset{n}{⟶}1$, almost surely.*
*(b)*  *If μ is ψ-mixing or summable ϕ-mixing, there exists $n_{1}\geq 1$ such that:*
  $$\underset{n\geq n_{1},x_{0}^{n-1}\in A^{n}}{\inf}\rho \left(x_{0}^{n-1}\right)=\rho_{-}>0\;.$$

If the alphabet, A is finite; the set $\left\{\rho \left(A\right):A\in A^{n},n<n_{1}\right\}$ is finite and has a strictly positive infimum, which implies that the infimum above can be taken over all n≥1.

## 4. Proofs of the Results

The statement of Theorem 1 is for ϕ and ψ and for hitting and return times. The case of return times under ϕ-mixing was already done by [6]. Our proof follows their method. In particular for the next subsection that lists a sequence of auxiliary results, some of them are not proven.

### 4.1. Preliminary Results

The following lemma plays a fundamental role in Theorems 1 and 2. It was originally proven in [25] assuming the summability of the function ϕ, an assumption that can be dropped.

**Lemma** **1.** *Let μ be a ϕ-mixing measure. Then, there exists positive constants C and c such that for all n≥1 and all $A\in A^{n}$, one has:*

$$\mu \left(A\right)\leq Ce^{-cn}.$$

**Proof.**  We denote by λ=sup{μ(a):a∈A}<1. Consider a positive integer $k_{0}$, and for all $n\geq k_{0}$, write $n=k_{0}q+r$, with 1≤q∈N and $0\leq r<k_{0}$. Suppose $A=a_{0}^{n-1}$, and apply the ϕ-mixing property to obtain:

$$\begin{array}{cc} \mu (A) & \leq \mu \left(\overset{q-1}{\underset{j=0}{⋂}}\left\{\sigma^{-jk_{0}}\left(a_{jk_{0}}\right)\right\}\right)\leq \mu \left(\overset{q-2}{\underset{j=0}{⋂}}\left\{\sigma^{-jk_{0}}\left(a_{jk_{0}}\right)\right\}\right)\left(\phi \left(k_{0}\right)+\mu \left(a_{\left(q-1\right)k_{0}}\right)\right)\\ \; & \leq \mu \left(\overset{q-2}{\underset{j=0}{⋂}}\left\{\sigma^{-jk_{0}}\left(a_{jk_{0}}\right)\right\}\right)\left(\phi \left(k_{0}\right)+\lambda \right). \end{array}$$

Iterating this argument, one concludes:

$$\mu \left(A\right)\leq {\left(\phi \left(k_{0}\right)+\lambda \right)}^{q}.$$

Since $\phi \left(k\right)\overset{k}{⟶}0$, there exists $k_{0}\in N$ such that $\phi (k_{0})+\lambda <1$. Thus, for $n\geq k_{0}$ and observing that $q=\frac{n-r}{k_{0}}>\frac{n}{k_{0}}-\frac{k_{0}-1}{k_{0}}$:

$$\mu \left(A\right)\leq {\left(\phi \left(k_{0}\right)+\lambda \right)}^{-\left(k_{0}-1\right)/k_{0}}{\left({\left(\phi (k_{0})+\lambda \right)}^{1/k_{0}}\right)}^{n}.$$

This covers the case $n\geq k_{0}$. By eventually enlarging the constant *C*, one covers the case $n<k_{0}$. This ends the proof. □

For a complete grammar, one has $\tau (A_{n}\left(x\right))\leq n$. Since we do not assume this, we need the following lemma, which provides upper bounds for τ(A) when μ is ψ-mixing or summable ϕ-mixing.

**Lemma** **2.** *Consider μ a ψ-mixing or summable ϕ-mixing measure. Then, there exists $n_{2}\in N$ such that for all $n\geq n_{2}$ and $A\in A^{n}$,*

- *τ(A)≤2n, for ψ;*
- *$\tau \left(A\right)\leq -\frac{2}{\mu \left(A\right)\ln\mu \left(A\right)}+n$, for summable ϕ.*

**Proof.**  We start with the case ψ. For *n* large enough, we have ψ(n)<1, which implies:

$$\mu \left(A\cap \sigma^{-2n}\left(A\right)\right)\geq \mu {\left(A\right)}^{2}\left(1-\psi \left(n\right)\right)>0$$

Since τ(A) is the smallest positive integer such that $\mu \left(A\cap \sigma^{-\tau (A)}\left(A\right)\right)>0$, one has τ(A)≤2n.

Now consider the ϕ-mixing case. The summability of ϕ ensures that for *g* large enough, we have ϕ(g)≤1/(glng). Thus:

$$\mu \left(A\cap \sigma^{-g-n}\left(A\right)\right)\geq \mu \left(A\right)\left(\mu \left(A\right)-\phi \left(g\right)\right)\geq \mu \left(A\right)\left(\mu \left(A\right)-\frac{1}{g\ln g}\right).$$

Take $g=-\frac{2}{\mu \left(A\right)\ln\mu \left(A\right)}$. The rightmost parenthesis above becomes:

$$\mu \left(A\right)\left[1-\frac{1}{2}\frac{-\ln\mu \left(A\right)}{\left(\ln\left(2\right)-\ln\mu \left(A\right)-\ln\left(-\ln\mu \left(A\right)\right)\right)}\right]$$

which is positive for *n* large enough. □

The multiplicative constant of two in both cases is technical and was chosen for the simplicity of the proof. Actually, it can be replaced by any constant strictly larger than one. An irreducible aperiodic finite state Markov chain with some entry equal to zero shows that this constant cannot be taken equal to one in the ψ-mixing case. Whether this bound is optimal for the ϕ-mixing case is an open question. Note that Lemmas 1 and 2 imply that $\tau \left(A\right)\mu \left(A\right)\overset{n}{⟶}0$ uniformly.

The remaining results of this subsection hold for $n\geq n^{\prime }$, where $n^{\prime }=1$ for the case of ϕ-mixing and:

(4) $$\begin{array}{c} n^{\prime }:=\inf\left\{n>2g_{0}:\psi \left(n\right)<1\right\} \end{array}$$

for the ψ-mixing case (see (1) for the definition of $g_{0}$).

Let us define:

$$M:=\psi \left(g_{0}+1\right)+1.$$

**Proposition** **1.** *Let μ be a ψ-mixing measure. Then, for all $n\geq n^{\prime }$, $A\in A^{n}$ and $k\geq n_{A}$, the following inequality holds:*

$$\mu_{A}\left(\tau \left(A\right)<T_{A}\leq k\right)\leq M\left(k-n_{A}+1\right)\mu \left(A^{\left(n_{A}-g_{0}\right)}\right).$$

**Proof.**  By definition of $n_{A}$, we first note that $\mu_{A}\left(\tau \left(A\right)<T_{A}\leq k\right)=\mu_{A}\left(n_{A}\leq T_{A}\leq k\right)$. Consider the case in which $n_{A}\leq n+g_{0}$. In this case, for $j\geq n_{A}$, one trivially has:

$$\left\{T_{A}=j\right\}\subset \sigma^{-j}\left(A\right)\subset \sigma^{-j-(n-\left(n_{A}-g_{0}\right))}\left(A^{\left(n_{A}-g_{0}\right)}\right).$$

Thus:

$$\begin{array}{c} \mu_{A}\left(n_{A}\leq T_{A}\leq k\right)\leq \mu_{A}\left(\underset{n_{A}\leq j\leq k}{⋃}\sigma^{-j-(n-\left(n_{A}-g_{0}\right))}\left(A^{\left(n_{A}-g_{0}\right)}\right)\right). \end{array}$$

Note that *A* and the union on the right hand-side in the above inequality are separated by a gap of length $g_{0}+1$. By ψ-mixing, one concludes that the left-hand side is bounded by:

$$\begin{array}{c} \left(\psi \left(g_{0}+1\right)+1\right)\mu \left(\underset{n_{A}\leq j\leq k}{⋃}\sigma^{-j-(n-\left(n_{A}-g_{0}\right))}\left(A^{\left(n_{A}-g_{0}\right)}\right)\right)\leq M\left(k-n_{A}+1\right)\mu \left(A^{\left(n_{A}-g_{0}\right)}\right). \end{array}$$

For $n_{A}>n+g_{0}$, recall first the convention in Section 3.2, which states that $\mu \left(A^{\left(n_{A}-g_{0}\right)}\right)=\mu \left(A\right)$. In a similar way to the first case,

$$\begin{array}{cc} \mu_{A}\left(n_{A}\leq T_{A}\leq k\right) & \leq \mu_{A}\left(\underset{n_{A}\leq j\leq k}{⋃}\sigma^{-j}\left(A\right)\right)\\ \; & \leq M\left(k-n_{A}+1\right)\mu \left(A\right). \end{array}$$

□

For the next proposition, recall that ϵ stands either for $\epsilon_{\psi }$ (2) or for $\epsilon_{\phi }$ (3), according to the mixing property of the measure under consideration. Further, let us use the notation ${T_{A}}^{\left[i\right]}:=T_{A}\circ \sigma^{i}$.

**Proposition** **2.** *Let μ be a ϕ or ψ-mixing measure. Then, for all $n\geq n^{\prime }$, $A\in A^{n}$ and t≥τ(A):*

$$|\mu_{A}\left(T_{A}>t\right)-\rho \left(A\right)\mu \left(T_{A}>t\right)|\leq C\epsilon \left(A\right),$$

*where C=4 for $\epsilon_{\phi }$ and C=4(M+1) for $\epsilon_{\psi }$.*

**Proof.**  The proof for $\epsilon_{\phi }$ can be found in Proposition 4.1 Item (b) of [6], and it remains valid even for a non-complete grammar and infinite alphabet. We observe that the error term defined therein is:

$$\epsilon^{\prime }\left(A\right)=\underset{1\leq w\leq n_{A}}{\inf}\left\{\left(2n+\tau \left(A\right)\right)\mu \left(A^{\left(w\right)}\right)+\phi \left(n_{A}-w+1\right))\right\}\leq 2\epsilon_{\phi }\left(A\right),$$

which justifies C=4 for this case.

Here, we prove the case $\epsilon_{\psi }$ in the same way. We start by assuming that t≥τ(A)+2n. By the triangle inequality:

$$\begin{array}{cc} \; & |\mu_{A}\left(T_{A}>t\right)-\rho \left(A\right)\mu \left(T_{A}>t\right)|\\ \; & \leq \left|\mu_{A}\left(T_{A}>\tau \left(A\right);{T_{A}}^{\left[\tau (A)\right]}>t-\tau \left(A\right)\right)-\right. \end{array}$$

(5) $$\begin{array}{cc} & \;\left.\mu_{A}\left(T_{A}>\tau \left(A\right);{T_{A}}^{\left[\tau (A)+2n\right]}>t-\tau \left(A\right)-2n\right)\right| \end{array}$$

(6) $$\begin{array}{cc} & +\left|\mu_{A}\left(T_{A}>\tau \left(A\right);{T_{A}}^{\left[\tau (A)+2n\right]}>t-\tau \left(A\right)-2n\right)-\rho \left(A\right)\mu \left(T_{A}>t-\tau \left(A\right)-2n\right)\right| \end{array}$$

(7) $$\begin{array}{cc} & +\left|\rho \left(A\right)\mu \left(T_{A}>t-\tau \left(A\right)-2n\right)-\rho \left(A\right)\mu \left(T_{A}>t\right)\right|. \end{array}$$

For the first modulus, by inclusion of sets:

$$\begin{array}{cc} (5)\; & \leq \mu_{A}\left(T_{A}>\tau \left(A\right);{T_{A}}^{\left[\tau (A)\right]}\leq 2n\right)=\mu_{A}\left(\tau \left(A\right)<T_{A}\leq \tau \left(A\right)+2n\right). \end{array}$$

If $n_{A}>\tau \left(A\right)+2n$, the last term is equal to zero. Otherwise, we apply Proposition 1 to obtain:

(8) $$\begin{array}{cc} \mu_{A}\left(\tau \left(A\right)<T_{A}\leq \tau \left(A\right)+2n\right) & \leq M\left(\tau \left(A\right)+2n-n_{A}+1\right)\mu \left(A^{\left(n_{A}-g_{0}\right)}\right)\\ & \leq 4Mn\mu \left(A^{\left(n_{A}-g_{0}\right)}\right) \end{array}$$

where the last inequality follows from Lemma 2.

By ψ-mixing, the modulus (6) is bounded by:

$$\rho \left(A\right)\mu (T_{A}>t-\tau \left(A\right)-2n)\psi \left(n\right)\leq \psi \left(n\right).$$

Note that the modulus is not needed for (7), and by inclusion:

$$\begin{array}{cc} \left(7\right) & \leq \rho \left(A\right)\mu \left({T_{A}}^{\left[t-\tau (A)-2n\right]}\leq \tau \left(A\right)+2n\right)\\ \; & =\rho \left(A\right)\mu \left(T_{A}\leq \tau \left(A\right)+2n\right)\\ \; & \leq (2n+\tau (A))\mu (A)\\ \; & \leq 4n\mu \left(A^{\left(n_{A}-g_{0}\right)}\right) \end{array}$$

where the equality and second inequality follow from the stationarity of μ.

Therefore, for t≥τ(A)+2n, the sum of (5), (6) and (7) is bounded by:

$$4Mn\mu \left(A^{\left(n_{A}-g_{0}\right)}\right)+\psi \left(n\right)+4n\mu \left(A^{\left(n_{A}-g_{0}\right)}\right)\leq 4\left(M+1\right)\epsilon_{\psi }\left(A\right).$$

We now consider the case where τ(A)≤t<τ(A)+2n. We have:

$$\begin{array}{cc} |\mu_{A}\left(T_{A}>t\right)-\rho \left(A\right)\mu \left(T_{A}>t\right)| & \leq |\mu_{A}\left(T_{A}>t\right)-\rho \left(A\right)|+|\rho \left(A\right)-\rho \left(A\right)\mu \left(T_{A}>t\right)|\\ \; & \leq \mu_{A}\left(\tau \left(A\right)<T_{A}\leq \tau \left(A\right)+2n\right)+t\mu \left(A\right)\\ \; & \leq 4Mn\mu \left(A^{\left(n_{A}-g_{0}\right)}\right)+\left(\tau \left(A\right)+2n\right)\mu \left(A^{\left(n_{A}-g_{0}\right)}\right)\\ \; & \leq 4\left(M+1\right)\epsilon_{\psi }\left(A\right). \end{array}$$

The lastinequality follows from (8). The other inequalities are straightforward. This ends the proof. □

The next lemma establishes upper bounds for the tail distribution at the scale given by Kac’s lemma, namely 1/μ(A). For technical reasons, we actually choose the scale:

$$f_{A}:=1/\left(2\mu \left(A\right)\right).$$

**Lemma** **3.** *Let μ be a stationary measure. Then, for all n≥1, $A\in A^{n}$, positive integer k, and $B\in F_{kf_{A}}^{\infty }$, the following inequalities hold:*

*(a)*  *$\mu \left(T_{A}>kf_{A};B\right)\leq {\left(\psi \left(n\right)+1\right)}^{k}\mu {\left(T_{A}>f_{A}-2n\right)}^{k}\mu \left(B\right)$,*
*(b)*  *$\mu \left(T_{A}>kf_{A};B\right)\leq {\left(\mu \left(T_{A}>f_{A}-2n\right)+\phi \left(n\right)\right)}^{k}\left(\mu \left(B\right)+\phi \left(n\right)\right)$,*
*(c)*  *$\mu_{A}\left(T_{A}>kf_{A};B\right)\leq {\left(\psi \left(n\right)+1\right)}^{k}\mu {\left(T_{A}>f_{A}-2n\right)}^{k-1}\mu \left(B\right)$.*

**Proof.**  We start by observing that $\left\{T_{A}>kf_{A}\right\}\subset \left\{T_{A}>kf_{A}-2n\right\}\in F_{0}^{kf_{A}-n}$. Thus, applying the ψ-mixing property, we get:

(9) $$\begin{array}{c} \mu \left(T_{A}>kf_{A};B\right)\leq \mu \left(T_{A}>kf_{A}-2n;B\right)\leq \left(\psi \left(n\right)+1\right)\mu \left(T_{A}>kf_{A}-2n\right)\mu \left(B\right) \end{array}$$

Furthermore:

$$\left\{T_{A}>kf_{A}-2n\right\}=\left\{T_{A}>\left(k-1\right)f_{A};{T_{A}}^{\left[\left(k-1\right)f_{A}\right]}>f_{A}-2n\right\}.$$

Now, one can take in particular $B=\left\{{T_{A}}^{\left[\left(k-1\right)f_{A}\right]}>f_{A}-2n\right\}\in F_{\left(k-1\right)f_{A}}^{\infty }$ and then apply (9) with k−1 instead of *k* to get:

$$\begin{array}{cc} \mu (T_{A}>kf_{A}-2n) & \leq \left(\psi \left(n\right)+1\right)\mu \left(T_{A}>\left(k-1\right)f_{A}-2n\right)\mu \left({T_{A}}^{\left[\left(k-1\right)f_{A}\right]}>f_{A}-2n\right)\\ \; & =\left(\psi \left(n\right)+1\right)\mu \left(T_{A}>\left(k-1\right)f_{A}-2n\right)\mu \left(T_{A}>f_{A}-2n\right). \end{array}$$

The equality follows by stationarity. Iterating this argument, one concludes that:

(10) $$\mu \left(T_{A}>kf_{A}-2n\right)\leq {\left(\psi \left(n\right)+1\right)}^{k-1}\mu {\left(T_{A}>f_{A}-2n\right)}^{k}\;.$$

Applying the resulting inequality in (9), we get Statement (a). In a similar way, ϕ-mixing gives:

$$\begin{array}{c} \mu \left(T_{A}>kf_{A};B\right)\leq \mu \left(T_{A}>kf_{A}-2n\right)\left(\mu \left(B\right)+\phi \left(n\right)\right). \end{array}$$

Thus:

(11) $$\begin{array}{cc} \mu (T_{A}>kf_{A}-2n)\; & \leq \mu \left(T_{A}>\left(k-1\right)f_{A}-2n\right)\left(\mu \left({T_{A}}^{\left[\left(k-1\right)f_{A}\right]}>f_{A}-2n\right)+\phi \left(n\right)\right)\\ & \leq {\left(\mu \left(T_{A}>f_{A}-2n\right)+\phi \left(n\right)\right)}^{k}. \end{array}$$

which ends the proof of (b).

The proof for (c) follows the same lines as Item (a), by observing that for $A,B\in F_{0}^{i}$ and $C\in F_{i+n}^{\infty }$, the ψ-mixing property implies $\mu_{A}\left(B;C\right)\leq \mu_{A}\left(B\right)\mu \left(C\right)\left(\psi \left(n\right)+1\right)$. □

The next proposition is the key to the proof of Theorem 1, and the idea is the following. We work under the time scale $f_{A}$. When $t=kf_{A},\;k\in N$, then we simply cut *t* into *k* pieces of equal size $f_{A}$. Then, the case of general $t=kf_{A}+r,r<f_{A}$ is approximated by its integer part $kf_{A}$. Technically, this is done in (b) and (a), respectively.

**Proposition** **3.** *Let μ be a ϕ or ψ-mixing measure. Then, for all $n\geq n^{\prime }$, $A\in A^{n}$ and positive integer k, the following inequalities hold:*

*(a)*  *For $0\leq r\leq f_{A}$:*

*1.*  $|\mu \left(T_{A}>kf_{A}+r\right)-\mu \left(T_{A}>kf_{A}\right)\mu \left(T_{A}>r\right)|\leq C^{\prime }{\left(\psi \left(n\right)+1\right)}^{k-1}\mu {\left(T_{A}>f_{A}-2n\right)}^{k}\epsilon_{\psi }\left(A\right)$
*2.*  $|\mu \left(T_{A}>kf_{A}+r\right)-\mu \left(T_{A}>kf_{A}\right)\mu \left(T_{A}>r\right)|\leq C^{\prime }{\left(\mu \left(T_{A}>f_{A}-2n\right)+\phi \left(n\right)\right)}^{k}\epsilon_{\phi }\left(A\right)$
*3.*  *$|\mu_{A}\left(T_{A}>kf_{A}+r\right)-\mu_{A}\left(T_{A}>kf_{A}\right)\mu \left(T_{A}>r\right)|\leq C^{\prime }{\left(\left(\psi \left(n\right)+1\right)\mu \left(T_{A}>f_{A}-2n\right)\right)}^{k-1}\epsilon_{\psi }\left(A\right)$.*

*(b)*  *For k≥1:*

*1.*  $\left|\mu \left(T_{A}>kf_{A}\right)-\mu {\left(T_{A}>f_{A}\right)}^{k}\right|\leq C^{\prime }\epsilon_{\psi }\left(A\right)\left(k-1\right){\left(\psi \left(n\right)+1\right)}^{k-2}\mu {\left(T_{A}>f_{A}-2n\right)}^{k-1}$
*2.*  $\left|\mu \left(T_{A}>kf_{A}\right)-\mu {\left(T_{A}>f_{A}\right)}^{k}\right|\leq C^{\prime }\epsilon_{\phi }\left(A\right)\left(k-1\right){\left(\mu \left(T_{A}>f_{A}-2n\right)+\phi \left(n\right)\right)}^{k-1}$
*3.*  $\left|\mu_{A}\left(T_{A}>kf_{A}\right)-\mu_{A}\left(T_{A}>f_{A}\right)\mu {\left(T_{A}>f_{A}\right)}^{k-1}\right|\leq C^{\prime }\epsilon_{\psi }\left(A\right)\left(k-1\right){\left(\left(\psi \left(n\right)+1\right)\mu \left(T_{A}>f_{A}-2n\right)\right)}^{k-2}$

*where $C^{\prime }=2\left(M+1\right)$ for the cases involving ψ and $C^{\prime }=4$ for ϕ.*

**Proof.**  We will prove Items (a)-1 and (a)-2 together. Initially, consider the case in which r<2n. In this case, for all $n\geq n^{\prime }$, we have:

(12) $$\begin{array}{cc} \; & |\mu \left(T_{A}>kf_{A}+r\right)-\mu \left(T_{A}>kf_{A}\right)\mu \left(T_{A}>r\right)|\\ \; & \leq \left|\mu \left(T_{A}>kf_{A},{T_{A}}^{\left[kf_{A}\right]}>r\right)-\mu \left(T_{A}>kf_{A}\right)\right|+\mu \left(T_{A}>kf_{A}\right)\left|1-\mu \left(T_{A}>r\right)\right|\\ \; & \leq \mu \left(T_{A}>kf_{A},{T_{A}}^{\left[kf_{A}\right]}\leq r\right)+\mu \left(T_{A}>kf_{A}\right)\mu \left(T_{A}\leq r\right). \end{array}$$

By Lemma 3-(a) and (10), the last sum is bounded by:

(13) $$\begin{array}{cc} \; & {\left(\left(\psi \left(n\right)+1\right)\mu \left(T_{A}>f_{A}-2n\right)\right)}^{k}\mu \left({T_{A}}^{\left[kf_{A}\right]}\leq r\right)+\mu \left(T_{A}>kf_{A}-2n\right)r\mu \left(A\right)\\ \; & \leq {\left(\left(\psi \left(n\right)+1\right)\mu \left(T_{A}>f_{A}-2n\right)\right)}^{k}\mu \left(T_{A}\leq r\right)+{\left(\psi \left(n\right)+1\right)}^{k-1}\mu {\left(T_{A}>f_{A}-2n\right)}^{k}r\mu \left(A\right)\\ \; & \leq {\left(\psi \left(n\right)+1\right)}^{k-1}\mu {\left(T_{A}>f_{A}-2n\right)}^{k}r\mu \left(A\right)\left(M+1\right)\\ \; & \leq 2\left(M+1\right){\left(\psi \left(n\right)+1\right)}^{k-1}\mu {\left(T_{A}>f_{A}-2n\right)}^{k}\epsilon_{\psi }\left(A\right) \end{array}$$

which gives us (a)-1. To get (a)-2 for r<2n, we apply Lemma 3-(b) and (11) in a similar way. Thus, (12) is bounded by:

$$\begin{array}{cc} \; & {\left(\mu \left(T_{A}>f_{A}-2n\right)+\phi \left(n\right)\right)}^{k}\left(\mu \left(T_{A}\leq r\right)+\phi \left(n\right)\right)+{\left(\mu \left(T_{A}>f_{A}-2n\right)+\phi \left(n\right)\right)}^{k}\mu \left(T_{A}\leq r\right)\\ \; & \leq 4{\left(\mu \left(T_{A}>f_{A}-2n\right)+\phi \left(n\right)\right)}^{k}\epsilon_{\phi }\left(A\right). \end{array}$$

We now consider the case r≥2n. The triangle inequality gives us:

$$\begin{array}{cc} \; & |\mu \left(T_{A}>kf_{A}+r\right)-\mu \left(T_{A}>kf_{A}\right)\mu \left(T_{A}>r\right)| \end{array}$$

(14) $$\begin{array}{cc} & \leq \left|\mu \left(T_{A}>kf_{A};{T_{A}}^{\left[kf_{A}\right]}>r\right)-\mu \left(T_{A}>kf_{A};{T_{A}}^{\left[kf_{A}+2n\right]}>r-2n\right)\right| \end{array}$$

(15) $$\begin{array}{cc} & +\left|\mu \left(T_{A}>kf_{A};{T_{A}}^{\left[kf_{A}+2n\right]}>r-2n\right)-\mu \left(T_{A}>kf_{A}\right)\mu \left({T_{A}}^{\left[kf_{A}+2n\right]}>r-2n\right)\right| \end{array}$$

(16) $$\begin{array}{cc} & +\left|\mu \left(T_{A}>kf_{A}\right)\mu \left({T_{A}}^{\left[kf_{A}+2n\right]}>r-2n\right)-\mu \left(T_{A}>kf_{A}\right)\mu \left(T_{A}>r\right)\right|. \end{array}$$

We proceed as in (5) and use Lemma 3-(a) to get:

(17) $$\begin{array}{cc} \left(14\right) & \leq \mu \left(T_{A}>kf_{A};{T_{A}}^{\left[kf_{A}\right]}\leq 2n\right)\\ \; & \leq (\left(\psi \left(n\right)+1\right){\left(\mu \left(T_{A}>f_{A}-2n\right)\right)}^{k}\mu \left(T_{A}\leq 2n\right)\\ \; & \leq 2n\mu \left(A\right)(\left(\psi \left(n\right)+1\right){\left(\mu \left(T_{A}>f_{A}-2n\right)\right)}^{k}. \end{array}$$

For the case ϕ, we apply Lemma 3-(b) and get:

(18) $$\begin{array}{cc} \left(14\right) & \leq {\left(\mu \left(T_{A}>f_{A}-2n\right)+\phi \left(n\right)\right)}^{k}\left(2n\mu \left(A\right)+\phi \left(n\right)\right). \end{array}$$

By ψ-mixing and (10):

(19) $$\begin{array}{cc} \left(15\right) & \leq \mu \left(T_{A}>kf_{A}-2n\right)\psi \left(n\right)\\ & \leq {\left(\psi \left(n\right)+1\right)}^{k-1}\mu {\left(T_{A}>f_{A}-2n\right)}^{k}\psi \left(n\right) \end{array}$$

Applying ϕ-mixing and (11):

(20) $$\begin{array}{cc} \left(15\right) & \leq {\left(\mu \left(T_{A}>f_{A}-2n\right)+\phi \left(n\right)\right)}^{k}\phi \left(n\right) \end{array}$$

Finally, using the shift-invariance and the same arguments as above:

(21) $$\begin{array}{cc} (16)\; & =\mu \left(T_{A}>kf_{A}\right)\mu \left(r-2n<T_{A}\leq r\right)\\ & \leq 2n\mu \left(A\right)\mu \left(T_{A}>kf_{A}-2n\right). \end{array}$$

Therefore, (10), (17), (19), and (21) give us:

$$\begin{array}{c} \left(14\right)+\left(15\right)+\left(16\right)\leq 2\left(M+1\right){\left(\psi \left(n\right)+1\right)}^{k-1}\mu {\left(T_{A}>f_{A}-2n\right)}^{k}\epsilon_{\psi }\left(A\right) \end{array}$$

and from (11), (18), (20), and (21), we get:

$$\begin{array}{c} \left(14\right)+\left(15\right)+\left(16\right)\leq 4{\left(\mu \left(T_{A}>f_{A}-2n\right)+\phi \left(n\right)\right)}^{k}\epsilon_{\phi }\left(A\right) \end{array}$$

which ends the proof of (a)-1 and (a)-2.

For the proof of (a)-3, we write a similar triangle inequality as above:

$$\begin{array}{cc} \; & |\mu_{A}\left(T_{A}>kf_{A}+r\right)-\mu_{A}\left(T_{A}>kf_{A}\right)\mu \left(T_{A}>r\right)|\\ \; & \leq \left|\mu_{A}\left(T_{A}>kf_{A};{T_{A}}^{\left[kf_{A}\right]}>r\right)-\mu_{A}\left(T_{A}>kf_{A};{T_{A}}^{\left[kf_{A}+2n\right]}>r-2n\right)\right|\\ \; & +\left|\mu_{A}\left(T_{A}>kf_{A};{T_{A}}^{\left[kf_{A}+2n\right]}>r-2n\right)-\mu_{A}\left(T_{A}>kf_{A}\right)\mu \left({T_{A}}^{\left[kf_{A}+2n\right]}>r-2n\right)\right|\\ \; & +\mu_{A}\left(T_{A}>kf_{A}\right)\left|\mu \left({T_{A}}^{\left[kf_{A}+2n\right]}>r-2n\right)-\mu \left(T_{A}>r\right)\right|. \end{array}$$

Then, we follow the same as we did for (a)-1, but applying Item (c) of Lemma 3 and using the ψ-mixing property:

$$|\mu_{A}\left(B;C\right)-\mu_{A}\left(B\right)\mu \left(C\right)|\leq \mu_{A}\left(B\right)\mu \left(C\right)\psi \left(n\right)$$

where $A,B\in F_{0}^{i}$ and $C\in F_{i+n}^{\infty }$. For the case r<2n, we use:

$$\begin{array}{cc} \; & |\mu_{A}\left(T_{A}>kf_{A}+r\right)-\mu_{A}\left(T_{A}>kf_{A}\right)\mu \left(T_{A}>r\right)|\\ \; & \leq \left|\mu_{A}\left(T_{A}>kf_{A},{T_{A}}^{\left[kf_{A}\right]}>r\right)-\mu_{A}\left(T_{A}>kf_{A}\right)\right|+\mu_{A}\left(T_{A}>kf_{A}\right)\left|1-\mu \left(T_{A}>r\right)\right| \end{array}$$

and proceed as we did in (13), applying again Lemma 3-(c). This ends Item (a).

We now come to the proof of Items (b)-1 and (b)-2. For k=1, we have an equality. For k≥2, we get:

(22) $$\begin{array}{cc} \; & \left|\mu \left(T_{A}>kf_{A}\right)-\mu {\left(T_{A}>f_{A}\right)}^{k}\right|\\ \; & =\left|\sum_{j=2}^{k}\left(\mu \left(T_{A}>jf_{A}\right)-\mu \left(T_{A}>\left(j-1\right)f_{A}\right)\mu \left(T_{A}>f_{A}\right)\right)\mu {\left(T_{A}>f_{A}\right)}^{k-j}\right|\\ & \leq \sum_{j=2}^{k}\left|\mu \left(T_{A}>jf_{A}\right)-\mu \left(T_{A}>\left(j-1\right)f_{A}\right)\mu \left(T_{A}>f_{A}\right)\right|\mu {\left(T_{A}>f_{A}\right)}^{k-j}. \end{array}$$

We put $r=f_{A}$ in Item (a)-1 to obtain (b)-1:

$$\begin{array}{cc} \left(22\right) & \leq 2\left(M+1\right)\epsilon_{\psi }\left(A\right)\sum_{j=2}^{k}{\left(\psi \left(n\right)+1\right)}^{j-2}\mu {\left(T_{A}>f_{A}-2n\right)}^{j-1}\mu {\left(T_{A}>f_{A}\right)}^{k-j}\\ \; & \leq 2\left(M+1\right)\epsilon_{\psi }\left(A\right)\left(k-1\right){\left(\psi \left(n\right)+1\right)}^{k-2}\mu {\left(T_{A}>f_{A}-2n\right)}^{k-1}. \end{array}$$

Furthermore, we get the inequality (b)-2, under ϕ-mixing, proceeding similarly as above:

$$\begin{array}{cc} \left(22\right) & \leq 4\epsilon_{\phi }\left(A\right)\sum_{j=2}^{k}{\left(\mu \left(T_{A}>f_{A}-2n\right)+\phi \left(n\right)\right)}^{j-1}{\left(\mu \left(T_{A}>f_{A}-2n\right)+\phi \left(n\right)\right)}^{k-j}\\ \; & =4\epsilon_{\phi }\left(A\right)\left(k-1\right){\left(\mu \left(T_{A}>f_{A}-2n\right)+\phi \left(n\right)\right)}^{k-1} \end{array}$$

Finally, we prove (b)-3 applying (a)-3 as follows:

$$\begin{array}{cc} \; & \left|\mu_{A}\left(T_{A}>kf_{A}\right)-\mu_{A}\left(T_{A}>f_{A}\right)\mu {\left(T_{A}>f_{A}\right)}^{k-1}\right|\\ \; & \leq \sum_{j=2}^{k}\left|\mu_{A}\left(T_{A}>jf_{A}\right)-\mu_{A}\left(T_{A}>\left(j-1\right)f_{A}\right)\mu \left(T_{A}>f_{A}\right)\right|\mu {\left(T_{A}>f_{A}\right)}^{k-j}\\ \; & \leq 2\left(M+1\right)\epsilon_{\psi }\left(A\right)\sum_{j=2}^{k}{\left(\left(\psi \left(n\right)+1\right)\mu \left(T_{A}>f_{A}-2n\right)\right)}^{j-2}\mu {\left(T_{A}>f_{A}\right)}^{k-j}\\ \; & \leq 2\left(M+1\right)\epsilon_{\psi }\left(A\right)\left(k-1\right){\left(\left(\psi \left(n\right)+1\right)\mu \left(T_{A}>f_{A}-2n\right)\right)}^{k-2}. \end{array}$$

□

The next two lemmas are classical results and are stated without proof. The first one establishes the reversibility of certain sets for stationary measures, and the second one is a discrete version of the mean value theorem, which follows with a straightforward computation.

**Lemma** **4.** *Let μ be a stationary measure. For all positive i∈N, n≥1 and $A\in A^{n}$:*

$$\mu \left(T_{A}=i\right)=\mu \left(T_{A}>i-1;A\right)$$

**Lemma** **5.** *Given $a_{1},\ldots ,a_{n},b_{1},\ldots ,b_{n}$ real numbers such that $0\leq a_{i},b_{i}\leq 1$, the following inequality holds:*

$$\begin{array}{c} \left|\prod_{i=1}^{n}a_{i}-\prod_{i=1}^{n}b_{i}\right|\leq \sum_{i=1}^{n}\left|a_{i}-b_{i}\right|{\left(\max_{1\leq i\leq n}\left\{a_{i},b_{i}\right\}\right)}^{n-1}\leq \sum_{i=1}^{n}\left|a_{i}-b_{i}\right|. \end{array}$$

### 4.2. Proof of Theorem 1

Theorem 1 contains eight statements, each statement corresponding to the choice of:

- recurrence time: hitting or return,
- mixing property: ψ or ϕ,
- amplitude of *t*: smaller or larger than $f_{A}$.

Recall the definition of $n^{\prime }$ in (4). The proof of Theorem 1 holds for all $n\geq n_{0}$, where $n_{0}$ is explicitly given by:

(23) $$\begin{array}{c} n_{0}:=\inf\left\{m\geq n^{\prime };\underset{A\in A^{n}}{\sup}\mu \left(A\right)\tau \left(A\right)<1/2,\;\forall n\geq m\right\} \end{array}$$

which is finite since $\sup_{A\in A^{n}}\mu \left(A\right)\tau \left(A\right)\overset{n}{⟶}0$. Then, in particular, we have $\tau \left(A\right)<f_{A}$ for all $n\geq n_{0}$ and $A\in A^{n}$.

#### 4.2.1. Proofs of the Statements for Small *t*’s

Here, we assume that $1\leq t\leq f_{A}:={\left[2\mu \left(A\right)\right]}^{-1}$.

**Proof of hitting time, ϕ and ψ together.**  Recall that ϵ(A) denotes $\epsilon_{\phi }\left(A\right)$ or $\epsilon_{\psi }\left(A\right)$, depending on whether the measure is ϕ or ψ-mixing.

Applying the inequality $\left|1-e^{-x}\right|\leq x$ for x≥0, we obtain the statement for 1≤t≤τ(A) as follows:

(24) $$\begin{array}{cc} \left|\mu \left(T_{A}>t\right)-e^{-\rho (A)\mu (A)t}\right| & \leq \left|\mu (T_{A}>t)-1\right|+\left|1-e^{-\rho (A)\mu (A)t}\right|\\ \; & \leq 2\tau (A)\mu (A). \end{array}$$

We consider now the case $\tau \left(A\right)<t\leq f_{A}$. For positive i∈N, define:

$$p_{i}=\frac{\mu_{A}\left(T_{A}>i-1\right)}{\mu (T_{A}>i-1)}.$$

Then:

(25) $$\begin{array}{cc} \frac{\mu (T_{A}>t)}{\mu (T_{A}>\tau \left(A\right))} & =\prod_{i=\tau (A)+1}^{t}\frac{\mu (T_{A}>i)}{\mu (T_{A}>i-1)}=\prod_{i=\tau (A)+1}^{t}\left(1-\mu (T_{A}=i|T_{A}>i-1)\right)\\ \; & =\prod_{i=\tau (A)+1}^{t}\left(1-\mu \left(A\right)p_{i}\right) \end{array}$$

where we used Lemma 4 in the last equality.

Thus, for $\tau \left(A\right)<t\leq f_{A}$, we apply (25) to obtain:

(26) $$\begin{array}{cc} \; & \;\left|\mu \left(T_{A}>t\right)-e^{-\rho (A)\mu (A)t}\right|\\ \; & =\left|\mu \left(T_{A}>\tau \left(A\right)\right)\prod_{i=\tau (A)+1}^{t}\left(1-\mu \left(A\right)p_{i}\right)-e^{-\rho (A)\mu (A)\tau (A)}\prod_{i=\tau (A)+1}^{t}e^{-\rho (A)\mu (A)}\right|\\ \; & \leq \left|\mu \left(T_{A}>\tau \left(A\right)\right)-e^{-\rho (A)\mu (A)\tau (A)}\right|+\left|\prod_{i=\tau (A)+1}^{t}\left(1-\mu \left(A\right)p_{i}\right)-\prod_{i=\tau (A)+1}^{t}e^{-\rho (A)\mu (A)}\right|\\ & \leq 2\tau \left(A\right)\mu \left(A\right)+\sum_{i=\tau (A)+1}^{t}\left|1-\mu \left(A\right)p_{i}-e^{-\rho (A)\mu (A)}\right| \end{array}$$

where the two inequalities follow from Lemma 5 and (24).

On the other hand, by the triangle inequality:

(27) $$\begin{array}{cc} \left|1-p_{i}\mu \left(A\right)-e^{-\rho (A)\mu (A)}\right| & \leq \left|p_{i}-\rho \left(A\right)\right|\mu \left(A\right)+\left|1-\rho \left(A\right)\mu \left(A\right)-e^{-\rho (A)\mu (A)}\right|. \end{array}$$

Since $|1-x-e^{-x}|\leq \frac{x^{2}}{2}$ for all 0≤x≤1, by doing x=ρ(A)μ(A), we get:

$$\begin{array}{c} \left|1-\rho \left(A\right)\mu \left(A\right)-e^{-\rho (A)\mu (A)}\right|\leq \frac{\rho {\left(A\right)}^{2}\mu {\left(A\right)}^{2}}{2}\leq \frac{\epsilon (A)\mu (A)}{2}. \end{array}$$

Furthermore, still for $\tau \left(A\right)+1\leq i\leq f_{A}+1$, Proposition 2 gives us:

$$\begin{array}{c} |p_{i}-\rho \left(A\right)|=\left|\frac{\mu_{A}\left(T_{A}>i-1\right)}{\mu (T_{A}>i-1)}-\rho \left(A\right)\right|\leq \frac{C\epsilon (A)}{\mu (T_{A}>i-1)}\leq 2C\epsilon \left(A\right), \end{array}$$

where, for the last inequality, we used:

$$\begin{array}{c} \mu \left(T_{A}>i-1\right)=1-\mu \left(T_{A}\leq i-1\right)\geq 1-\left(i-1\right)\mu \left(A\right)\geq 1-f_{A}\mu \left(A\right)=\frac{1}{2}. \end{array}$$

Thus, applying (27), we obtain for $\tau \left(A\right)+1\leq i\leq f_{A}+1$:

(28) $$\begin{array}{c} \left|1-p_{i}\mu \left(A\right)-e^{-\rho (A)\mu (A)}\right|\leq \left(2C+1/2\right)\epsilon \left(A\right)\mu \left(A\right). \end{array}$$

Therefore, (26) and (28) give us:

(29) $$\begin{array}{cc} \left|\mu \left(T_{A}>t\right)-e^{-\rho (A)\mu (A)t}\right| & \leq 2\tau \left(A\right)\mu \left(A\right)+\left(2C+1/2\right)\left(t-\tau \left(A\right)\right)\epsilon \left(A\right)\mu \left(A\right)\\ & \leq \left(2C+1/2\right)\left[\tau \left(A\right)\mu \left(A\right)+t\mu \left(A\right)\epsilon \left(A\right)\right] \end{array}$$

which concludes the statement of Theorem 1 for hitting time at small *t*’s (with either ϕ or ψ). □

**Proof for return time, ϕ and ψ together.**  We first note that the statement is trivial for t=τ(A), then we consider t>τ(A). By definition, we have $\mu_{A}\left(T_{A}>t\right)=p_{t+1}\mu \left(T_{A}>t\right)$. Then, we use again the triangle inequality to write:

(30) $$\begin{array}{cc} \; & \;\left|\mu_{A}\left(T_{A}>t\right)-\rho \left(A\right)e^{-\rho (A)\mu (A)(t-\tau (A))}\right|\\ & \leq \mu \left(T_{A}>t\right)\left|p_{t+1}-\rho \left(A\right)\right|+\rho \left(A\right)\left|\mu \left(T_{A}>t\right)-e^{-\rho (A)\mu (A)(t-\tau (A))}\right|. \end{array}$$

As we saw before, the first modulus above is bounded by 2Cϵ(A). On the other hand, we apply (25) to obtain for $\tau \left(A\right)<t\leq f_{A}$:

$$\begin{array}{c} \left|\mu \left(T_{A}>t\right)-e^{-\rho (A)\mu (A)(t-\tau (A))}\right|=\left|\mu \left(T_{A}>\tau \left(A\right)\right)\prod_{i=\tau (A)+1}^{t}\left(1-\mu \left(A\right)p_{i}\right)-\prod_{i=\tau (A)+1}^{t}e^{-\rho (A)\mu (A)}\right|. \end{array}$$

This is bounded, applying Lemma 5, by:

$$\begin{array}{cc} \; & \;\;|\mu \left(T_{A}>\tau \left(A\right)\right)-1|+\left|\prod_{i=\tau (A)+1}^{t}\left(1-\mu \left(A\right)p_{i}\right)-\prod_{i=\tau (A)+1}^{t}e^{-\rho (A)\mu (A)}\right|\\ \; & \leq \tau \left(A\right)\mu \left(A\right)+\left(2C+1/2\right)t\mu \left(A\right)\epsilon \left(A\right) \end{array}$$

where the last inequality follows from (26) and (28). Finally, notice that $t\mu \left(A\right)\leq f_{A}\mu \left(A\right)=1/2$ and τ(A)μ(A)≤2ϵ(A) (use Lemma 2 for ψ). Therefore, we obtain from (30):

(31) $$\begin{array}{c} \left|\mu_{A}\left(T_{A}>t\right)-\rho \left(A\right)e^{-\rho (A)\mu (A)(t-\tau (A))}\right|\leq \left(3C+9/4\right)\epsilon \left(A\right) \end{array}$$

This concludes the statement of Theorem 1 for the return time at small *t*’s (with either ϕ or ψ). □

#### 4.2.2. Proof of the Statements for Large *t*’s

The proof for the return time for $t>f_{A}$ was given in [6] under ϕ-mixing, a finite alphabet, and a complete grammar. The proof still holds if one just assumes a countable alphabet and an incomplete grammar (recall Remark 2 for the uniform convergence to zero of the error term $\epsilon_{\phi }$). Thus, we focus on the hitting time under each mixing assumption and the return time only under ψ-mixing.

**Proof of Theorem 1 for hitting times, for $t>f_{A}$.**  Write $t=kf_{A}+r$ with integer k≥1 and $0\leq r<f_{A}$. Thus, we have:

(32) $$\begin{array}{cc} \left|\mu \left(T_{A}>t\right)-e^{-\rho (A)\mu (A)t}\right| & \leq \left|\mu \left(T_{A}>kf_{A}+r\right)-\mu \left(T_{A}>kf_{A}\right)\mu \left(T_{A}>r\right)\right| \end{array}$$

(33) $$\begin{array}{cc} & \;+\left|\mu \left(T_{A}>kf_{A}\right)-\mu {\left(T_{A}>f_{A}\right)}^{k}\right|\mu \left(T_{A}>r\right) \end{array}$$

(34) $$\begin{array}{cc} & \;+\left|\mu {\left(T_{A}>f_{A}\right)}^{k}-e^{-\rho \left(A\right)\frac{k}{2}}\right|\mu \left(T_{A}>r\right) \end{array}$$

(35) $$\begin{array}{cc} & \;+\left|e^{-\rho \left(A\right)\frac{k}{2}}\mu \left(T_{A}>r\right)-e^{-\rho (A)\mu (A)t}\right|. \end{array}$$

In order to get an upper bound for the sum of (32) and (33), we analyse the ψ and ϕ cases separately and start with the ψ-mixing. Applying Items (a)-1 and (b)-1 of Proposition 3, that sum is bounded by:

(36) $$\begin{array}{cc} \; & \leq C^{\prime }\epsilon_{\psi }\left(A\right){\left(\psi \left(n\right)+1\right)}^{k-1}\mu {\left(T_{A}>f_{A}-2n\right)}^{k}\left(1+\left(k-1\right){\left(\left(\psi \left(n\right)+1\right)\mu \left(T_{A}>f_{A}-2n\right)\right)}^{-1}\right)\\ \; & \leq 2\left(M+1\right)\epsilon_{\psi }\left(A\right){\left(\left(\psi \left(n\right)+1\right)\mu \left(T_{A}>f_{A}-2n\right)\right)}^{k}2k\\ & \leq 8\left(M+1\right)\epsilon_{\psi }\left(A\right)\mu \left(A\right)t{\left(\left(\psi \left(n\right)+1\right)\mu \left(T_{A}>f_{A}-2n\right)\right)}^{k}. \end{array}$$

where the last two inequalities are justified by $\mu {\left(T_{A}>f_{A}-2n\right)}^{-1}\leq \mu {\left(T_{A}>f_{A}\right)}^{-1}\leq 2$ and k≤2μ(A)t.

On the other hand, applying (29) with $t=f_{A}-2n$, we get:

$$\begin{array}{cc} \left|\mu \left(T_{A}>f_{A}-2n\right)-e^{-\rho \left(A\right)\mu \left(A\right)\left(f_{A}-2n\right)}\right| & =\left|\mu \left(T_{A}>f_{A}-2n\right)-e^{-\frac{\rho (A)}{2}+2n\rho \left(A\right)\mu \left(A\right)}\right|\\ \; & \leq \left(2C+1/2\right)\left(\tau \left(A\right)\mu \left(A\right)+\left(f_{A}-2n\right)\mu \left(A\right)\epsilon_{\psi }\left(A\right)\right)\\ \; & \leq \left(5C+5/4\right)\epsilon_{\psi }\left(A\right) \end{array}$$

where we use $\tau \left(A\right)\mu \left(A\right)\leq 2\epsilon_{\psi }\left(A\right)$.

Furthermore, by the Mean Value Theorem (MVT):

$$\begin{array}{cc} \left|e^{-\frac{\rho (A)}{2}+2n\rho \left(A\right)\mu \left(A\right)}-e^{-\frac{\rho (A)}{2}}\right| & \leq 2n\rho \left(A\right)\mu \left(A\right)e^{-\frac{\rho (A)}{2}+2n\rho \left(A\right)\mu \left(A\right)}\\ \; & \leq 2n\mu \left(A\right)e^{2n\mu (A)}\leq \frac{11}{2}n\mu \left(A\right) \end{array}$$

since for $n\geq n_{0}$, we have 2nμ(A)≤2supμ(A)τ(A)≤1.

Thus, it follows that:

$$\begin{array}{cc} \; & \;\left|\left(\psi \left(n\right)+1\right)\mu \left(T_{A}>f_{A}-2n\right)-e^{-\frac{\rho (A)}{2}}\right|\\ \; & \leq \psi \left(n\right)+\left|\mu \left(T_{A}>f_{A}-2n\right)-e^{-\frac{\rho (A)}{2}+2n\rho \left(A\right)\mu \left(A\right)}\right|+\left|e^{-\frac{\rho (A)}{2}+2n\rho \left(A\right)\mu \left(A\right)}-e^{-\frac{\rho (A)}{2}}\right|\\ \; & \leq \left(5C+27/4\right)\epsilon_{\psi }\left(A\right). \end{array}$$

Therefore:

$${\left(\left(\psi \left(n\right)+1\right)\mu \left(T_{A}>f_{A}-2n\right)\right)}^{k}\leq {\left(e^{-\frac{\rho (A)}{2}}+\left(5C+27/4\right)\epsilon_{\psi }\left(A\right)\right)}^{k}.$$

Since $e^{x}-1\geq x\;\forall x\in R$, by doing $K=\left(5C+27/4\right)e^{1/2}$, we get:

(37) $$\begin{array}{cc} \; & \left(e^{K\epsilon_{\psi }\left(A\right)}-1\right)\geq K\epsilon_{\psi }\left(A\right)\geq \left(5C+27/4\right)\epsilon_{\psi }\left(A\right)e^{\frac{\rho (A)}{2}}\\ ⟹ & \;e^{-\frac{\rho (A)}{2}}\left(e^{K\epsilon_{\psi }\left(A\right)}-1\right)\geq \left(5C+27/4\right)\epsilon_{\psi }\left(A\right)\\ ⟹ & \;e^{-\frac{\rho (A)}{2}+K\epsilon_{\psi }\left(A\right)}\geq \left(5C+27/4\right)\epsilon_{\psi }\left(A\right)+e^{-\frac{\rho (A)}{2}}. \end{array}$$

Now, using that k=2μ(A)(t−r), we have:

(38) $$\begin{array}{cc} {\left(\left(\psi \left(n\right)+1\right)\mu \left(T_{A}>f_{A}-2n\right)\right)}^{k} & \leq {\left(e^{-\frac{\rho (A)}{2}+K\epsilon_{\psi }\left(A\right)}\right)}^{k}\\ \; & =e^{-\rho \left(A\right)\mu \left(A\right)t+\rho \left(A\right)\mu \left(A\right)r+2K\epsilon_{\psi }\left(A\right)\mu \left(A\right)t-2K\epsilon_{\psi }\left(A\right)\mu \left(A\right)r}\\ \; & \leq e^{-\mu \left(A\right)t\left(\rho \left(A\right)-2K\epsilon_{\psi }\left(A\right)\right)}e^{\mu (A)r}\\ & \leq e^{1/2}e^{-\mu \left(A\right)t\left(\rho \left(A\right)-C_{3}\epsilon_{\psi }\left(A\right)\right)} \end{array}$$

where the last inequality follows from $e^{\mu (A)r}\leq e^{\mu \left(A\right)f_{A}}$.

Therefore, it follows from (36) that the sum of (32) and (33) is bounded by:

$$\begin{array}{c} 14\left(M+1\right)\epsilon_{\psi }\left(A\right)\mu \left(A\right)te^{-\mu \left(A\right)t\left(\rho \left(A\right)-C_{3}\epsilon_{\psi }\left(A\right)\right)}. \end{array}$$

We now turn to the case of ϕ-mixing. We apply Items (a)-2 and (b)-2 of Proposition 3 to get an upper bound for the sum of (32) and (33):

$$\begin{array}{cc} \; & \left|\mu \left(T_{A}>kf_{A}+r\right)-\mu \left(T_{A}>kf_{A}\right)\mu \left(T_{A}>r\right)\right|+\left|\mu \left(T_{A}>kf_{A}\right)-\mu {\left(T_{A}>f_{A}\right)}^{k}\right|\mu \left(T_{A}>r\right)\\ \; & \leq 4\epsilon_{\phi }\left(A\right){\left(\mu \left(T_{A}>f_{A}-2n\right)+\phi \left(n\right)\right)}^{k}\left(1+\left(k-1\right){\left(\mu \left(T_{A}>f_{A}-2n\right)+\phi \left(n\right)\right)}^{-1}\right)\\ \; & \leq 4\epsilon_{\phi }\left(A\right){\left(\mu \left(T_{A}>f_{A}-2n\right)+\phi \left(n\right)\right)}^{k}2k\\ \; & \leq 16\epsilon_{\phi }\left(A\right)\mu \left(A\right)t{\left(\mu \left(T_{A}>f_{A}-2n\right)+\phi \left(n\right)\right)}^{k}. \end{array}$$

Similarly to the ψ-mixing case, one obtains:

$$\begin{array}{c} {\left(\mu \left(T_{A}>f_{A}-2n\right)+\phi \left(n\right)\right)}^{k}\leq e^{1/2}e^{-\mu \left(A\right)t\left(\rho \left(A\right)-C_{3}\epsilon_{\phi }\left(A\right)\right)} \end{array}$$

which implies in the ϕ-mixing case that the sum of (32) and (33) is bounded by:

$$27\epsilon_{\phi }\left(A\right)\mu \left(A\right)te^{-\mu \left(A\right)t\left(\rho \left(A\right)-C_{3}\epsilon_{\phi }\left(A\right)\right)}.$$

Now, we will treat the cases ψ and ϕ together to obtain upper bounds for (34) and (35). In order to get an upper bound for (34), we apply (29) with $t=f_{A}$:

(39) $$\begin{array}{cc} \left|\mu \left(T_{A}>f_{A}\right)-e^{-\rho \left(A\right)\mu \left(A\right)f_{A}}\right| & =\left|\mu \left(T_{A}>f_{A}\right)-e^{-\frac{\rho (A)}{2}}\right|\\ \; & \leq \left(2C+1/2\right)\left(\tau \left(A\right)\mu \left(A\right)+f_{A}\mu \left(A\right)\epsilon \left(A\right)\right)\\ & \leq \left(5C+5/4\right)\epsilon \left(A\right). \end{array}$$

Thus, applying Lemma 5, we have:

$$\begin{array}{cc} \; & \;\left|\mu {\left(T_{A}>f_{A}\right)}^{k}-e^{-\rho \left(A\right)\frac{k}{2}}\right|\\ \; & \leq \sum_{i=1}^{k}\left|\mu \left(T_{A}>f_{A}\right)-e^{-\frac{\rho (A)}{2}}\right|{\left(\max\left\{\mu \left(T_{A}>f_{A}\right),e^{-\frac{\rho (A)}{2}}\right\}\right)}^{k-1}. \end{array}$$

The max is bounded using (39) by:

$$\begin{array}{c} e^{-\frac{\rho (A)}{2}}+\left(5C+5/4\right)\epsilon \left(A\right). \end{array}$$

Naturally, the absolute value is also bounded by using (39), and we get that the above sum is bounded above by:

(40) $$\begin{array}{c} k\;\left(5C+5/4\right)\epsilon \left(A\right)\;{\left(e^{-\frac{\rho (A)}{2}}+\left(5C+5/4\right)\epsilon \left(A\right)\right)}^{k-1}. \end{array}$$

Recalling that k=2μ(A)(t−r) and proceeding as we did for (37) and (38), we get the following upper bound for (34):

$$2\left(5C+5/4\right)\epsilon \left(A\right)\mu \left(A\right)t\;e^{-\mu \left(A\right)t\left(\rho \left(A\right)-C_{3}\epsilon \left(A\right)\right)}e^{1}\leq 7\left(4C+1\right)\epsilon \left(A\right)\mu \left(A\right)te^{-\mu \left(A\right)t\left(\rho \left(A\right)-C_{3}\epsilon \left(A\right)\right)}.$$

To conclude the proof for the hitting time, we apply (29) with t=r to bound (35) as follows:

$$\begin{array}{cc} \left|e^{-\rho \left(A\right)\frac{k}{2}}\mu \left(T_{A}>r\right)-e^{-\rho (A)\mu (A)t}\right| & =e^{-\rho (A)\mu (A)t+\rho (A)\mu (A)r}\left|\mu \left(T_{A}>r\right)-e^{-\rho (A)\mu (A)r}\right|\\ \; & \leq \left(2C+1/2\right)e^{-\rho \left(A\right)\mu \left(A\right)t+\mu \left(A\right)f_{A}}\left(\tau (A)\mu (A)+r\mu (A)\epsilon (A)\right)\\ \; & \leq \left(2C+1/2\right)\left(\tau \left(A\right)\mu \left(A\right)+f_{A}\mu \left(A\right)\epsilon \left(A\right)\right)e^{-\rho (A)\mu (A)t}e^{1/2}\\ \; & \leq \left(17C+5\right)\epsilon \left(A\right)\mu \left(A\right)te^{-\mu \left(A\right)t\left(\rho \left(A\right)-C_{3}\epsilon \left(A\right)\right)} \end{array}$$

where the term μ(A)t follows from $1=2\mu \left(A\right)f_{A}\leq 2\mu \left(A\right)t$. □

**Proof of Theorem 1 for the return time, for $t>f_{A}$ and under ψ-mixing.**  We use again the triangle inequality to write:

$$\begin{array}{cc} \; & \left|\mu_{A}\left(T_{A}>t\right)-\rho \left(A\right)e^{-\rho (A)\mu (A)(t-\tau (A))}\right| \end{array}$$

(41) $$\begin{array}{cc} & \leq \left|\mu_{A}\left(T_{A}>kf_{A}+r\right)-\mu_{A}\left(T_{A}>kf_{A}\right)\mu \left(T_{A}>r\right)\right| \end{array}$$

(42) $$\begin{array}{cc} & +\left|\mu_{A}\left(T_{A}>kf_{A}\right)-\mu_{A}\left(T_{A}>f_{A}\right)\mu {\left(T_{A}>f_{A}\right)}^{k-1}\right|\mu \left(T_{A}>r\right) \end{array}$$

(43) $$\begin{array}{cc} & +\left|\mu_{A}\left(T_{A}>f_{A}\right)\mu {\left(T_{A}>f_{A}\right)}^{k-1}-\rho \left(A\right)e^{-\rho \left(A\right)\frac{k}{2}}\right|\mu \left(T_{A}>r\right) \end{array}$$

(44) $$\begin{array}{cc} & +\rho \left(A\right)e^{-\rho \left(A\right)\frac{k}{2}}\left|\mu \left(T_{A}>r\right)-e^{-\rho (A)\mu (A)(r-\tau (A))}\right|. \end{array}$$

Applying Items (a)-3 and (b)-3 of Proposition 3, the sum of (41) and (42) is bounded by:

$$\begin{array}{cc} \; & 2\left(M+1\right)\epsilon_{\psi }\left(A\right){\left(\left(\psi \left(n\right)+1\right)\mu \left(T_{A}>f_{A}-2n\right)\right)}^{k-1}\left(1+\left(k-1\right){\left(\left(\psi \left(n\right)+1\right)\mu \left(T_{A}>f_{A}-2n\right)\right)}^{-1}\right)\\ \; & \leq 2\left(M+1\right)\epsilon_{\psi }\left(A\right){\left(\left(\psi \left(n\right)+1\right)\mu \left(T_{A}>f_{A}-2n\right)\right)}^{k-1}2k\\ \; & \leq 8\left(M+1\right)\epsilon_{\psi }\left(A\right)\mu \left(A\right)t{\left(\left(\psi \left(n\right)+1\right)\mu \left(T_{A}>f_{A}-2n\right)\right)}^{k-1}. \end{array}$$

Replacing *k* by k−1 in (38), the last term is bounded above by:

$$8\left(M+1\right)\epsilon_{\psi }\left(A\right)\mu \left(A\right)t\;e^{1}\;e^{-\mu \left(A\right)t\left(\rho \left(A\right)-C_{3}\epsilon_{\psi }\left(A\right)\right)}\leq 22\left(M+1\right)\epsilon_{\psi }\left(A\right)\mu \left(A\right)t\;e^{-\mu \left(A\right)t\left(\rho \left(A\right)-C_{3}\epsilon_{\psi }\left(A\right)\right)}.$$

On the other hand, Lemma 5 gives us:

$$\begin{array}{cc} \left(43\right) & \leq {\left(\max\left\{\mu_{A}\left(T_{A}>f_{A}\right),\mu \left(T_{A}>f_{A}\right),e^{-\rho (A)/2}\right\}\right)}^{k-1}\left(\left|\mu_{A}\left(T_{A}>f_{A}\right)-\rho \left(A\right)e^{-\rho (A)/2}\right|\right.\\ \; & \left.+\sum_{i=1}^{k-1}\left|\mu \left(T_{A}>f_{A}\right)-e^{-\rho (A)/2}\right|\right) \end{array}$$

The last sum is bounded by $\left(5C+5/4\right)\left(k-1\right)\epsilon_{\psi }\left(A\right)$ using (39). On the other hand, applying (31) with $t=f_{A}$ and the MVT, we obtain:

$$\begin{array}{cc} \; & \left|\mu_{A}\left(T_{A}>f_{A}\right)-\rho \left(A\right)e^{-\rho (A)/2}\right|\\ \; & \leq \left|\mu_{A}\left(T_{A}>f_{A}\right)-\rho \left(A\right)e^{-\rho (A)/2+\rho (A)\mu (A)\tau (A)}\right|+\rho \left(A\right)\left|e^{-\rho (A)/2+\rho (A)\mu (A)\tau (A)}-e^{-\rho (A)/2}\right|\\ \; & \leq \left(3C+9/4\right)\epsilon_{\psi }\left(A\right)+\rho \left(A\right)\mu \left(A\right)\tau \left(A\right)e^{-\rho (A)(1/2-\mu (A)\tau (A))}\\ \; & \leq \left(3C+17/4\right)\epsilon_{\psi }\left(A\right) \end{array}$$

since $e^{-\rho (A)(1/2-\mu (A)\tau (A))}\leq 1$ and $\mu \left(A\right)\tau \left(A\right)\leq 2\epsilon_{\psi }\left(A\right)$ for $n\geq n_{0}$.

Furthermore, the last inequality implies:

$$\mu_{A}\left(T_{A}>f_{A}\right)\leq \rho \left(A\right)e^{-\rho (A)/2}+\left(3C+17/4\right)\epsilon_{\psi }\left(A\right)\leq e^{-\rho (A)/2}+\left(5C+5/4\right)\epsilon_{\psi }\left(A\right)$$

and by (39), we get:

$$\max\left\{\mu_{A}\left(T_{A}>f_{A}\right),\mu \left(T_{A}>f_{A}\right),e^{-\rho (A)/2}\right\}\leq e^{-\rho (A)/2}+\left(5C+5/4\right)\epsilon_{\psi }\left(A\right).$$

Therefore, as we saw in (40), we have:

$$\begin{array}{cc} \left(43\right) & \leq \left(5C+5/4\right)\epsilon_{\psi }\left(A\right)k{\left(e^{-\rho (A)/2}+\left(5C+5/4\right)\epsilon_{\psi }\left(A\right)\right)}^{k-1}\\ \; & \leq 2\left(5C+5/4\right)\epsilon_{\psi }\left(A\right)\mu \left(A\right)t\;e^{1}\;e^{-\mu \left(A\right)t\left(\rho \left(A\right)-C_{3}\epsilon_{\psi }\left(A\right)\right)}\\ \; & \leq \left(109M+116\right)\epsilon_{\psi }\left(A\right)\mu \left(A\right)te^{-\mu \left(A\right)t\left(\rho \left(A\right)-C_{3}\epsilon_{\psi }\left(A\right)\right)}. \end{array}$$

Finally, by doing t=r in (29) and applying the MVT once again, we get:

$$\begin{array}{cc} \; & \left|\mu \left(T_{A}>r\right)-e^{-\rho (A)\mu (A)(r-\tau (A))}\right|\\ \; & \leq \left|\mu \left(\tau_{A}>r\right)-e^{-\rho (A)\mu (A)r}\right|+\left|e^{-\rho (A)\mu (A)r}-e^{-\rho (A)\mu (A)(r-\tau (A))}\right|\\ \; & \leq \left(2C+1/2\right)\left(\tau \left(A\right)\mu \left(A\right)+r\mu \left(A\right)\epsilon_{\psi }\left(A\right)\right)+\rho \left(A\right)\mu \left(A\right)\tau \left(A\right)e^{-\rho (A)\mu (A)(r-\tau (A))}\\ \; & \leq \left(2C+1/2\right)\left(2\epsilon_{\psi }\left(A\right)+f_{A}\mu \left(A\right)\epsilon_{\psi }\left(A\right)\right)+\left(7/2\right)\epsilon_{\psi }\left(A\right)\\ \; & \leq \left(5C+19/4\right)\epsilon_{\psi }\left(A\right). \end{array}$$

The third inequality follows from $e^{-\rho (A)\mu (A)(r-\tau (A))}\leq e^{\rho (A)\mu (A)\tau (A)}\leq e^{1/2}$, since $n\geq n_{0}$. Now, just note that $\rho \left(A\right)\mu \left(A\right)\tau \left(A\right)\leq 2\epsilon_{\psi }\left(A\right)$.

Therefore, we finish the proof by obtaining the following upper bound:

$$\begin{array}{cc} \left(44\right) & \leq \left(5C+19/4\right)\epsilon_{\psi }\left(A\right)e^{\rho (A)\mu (A)r}e^{-\rho (A)\mu (A)t}\\ \; & \leq \left(5C+19/4\right)\epsilon_{\psi }\left(A\right)2\mu \left(A\right)t\;e^{f_{A}\mu \left(A\right)}e^{-\mu \left(A\right)t(\rho \left(A\right)-C_{3}\epsilon_{\psi }\left(A\right))}\\ \; & \leq \left(66M+82\right)\epsilon_{\psi }\left(A\right)\mu \left(A\right)te^{-\mu \left(A\right)t(\rho \left(A\right)-C_{3}\epsilon_{\psi }\left(A\right))}. \end{array}$$

□

### 4.3. Proof of Theorem 2

**Proof of Statement** **(a).** For each x∈X, we define:

$$\tau \left(x\right):=\sup\{\tau \left(x_{0}^{n-1}\right),n\geq 1\}.$$

Let B={x∈X;τ(x)=∞} be the set of aperiodic points of X. For x∈B, denote $A_{n}=A_{n}\left(x\right)$, and consider the case $\tau (A_{n})<n$. Then, we have:

$$\begin{array}{cc} 1-\rho (A_{n}) & =\mu_{A_{n}}\left(T_{A_{n}}=\tau \left(A_{n}\right)\right)\\ \; & =\mu_{A_{n}}\left(\sigma^{-n}\left(A_{n}^{(\tau \left(A_{n}\right)}\right)\right)\\ \; & \leq \mu_{A_{n}}\left(\sigma^{-n-\lfloor \tau \left(A_{n}\right)/2\rfloor }\left(A_{n}^{(\left\lceil \tau \left(A_{n}\right)/2\right\rceil }\right)\right)\\ \; & \leq \mu \left(A_{n}^{(\left\lceil \tau \left(A_{n}\right)/2\right\rceil }\right)+\phi \left(\lfloor \tau \left(A_{n}\right)/2\rfloor +1\right). \end{array}$$

Since x∈B, we have $\tau \left(A_{n}\right)\overset{n}{⟶}\infty$, which implies that the last expression converges to zero. For the case $\tau (A_{n})\geq n$, we use the same argument:

$$\begin{array}{cc} 1-\rho (A_{n}) & =\mu_{A_{n}}\left(\sigma^{-\tau (A_{n})}\left(A_{n}\right)\right)\\ \; & \leq \mu_{A_{n}}\left(\sigma^{-\tau \left(A_{n}\right)-\left\lfloor n/2\right\rfloor }\left(A_{n}^{\left(\lceil n/2\right\rceil }\right)\right)\\ \; & \leq \mu \left(A_{n}^{\left(\lceil n/2\rceil \right)}\right)+\phi \left(\lfloor n/2\rfloor +1\right) \end{array}$$

which also converges to zero. Therefore, $\rho \left(A_{n}\right)\overset{n}{⟶}1$. We conclude the proof by noting that X−B is a countable set, and thus, μ(B)=1. □

**Proof of Statement** **(b).** By Lemma 2, for ψ-mixing or summable ϕ-mixing measures, there exists $n_{0}\geq 1$ such that:

$$\forall n\geq n_{0},\;\forall A\in A^{n},\;\;\mu {\left(A\right)}^{-1}>\tau \left(A\right)\;.$$

Now, since $\mu_{A}\left(T_{A}>j\right),j\geq 1$ is a nonincreasing sequence, the potential well is larger than or equal to the arithmetic mean of the subsequent $\mu {\left(A\right)}^{-1}-\tau \left(A\right)$ elements:

(45) $$\begin{array}{cc} \rho \left(A\right)=\mu_{A}\left(T_{A}>\tau \left(A\right)\right)\; & \geq \frac{1}{\mu {\left(A\right)}^{-1}-\tau \left(A\right)}\sum_{j=\tau (A)}^{\mu {\left(A\right)}^{-1}-1}\mu_{A}\left(T_{A}>j\right)\\ \; & \geq \frac{1}{\mu {\left(A\right)}^{-1}}\sum_{j=\tau (A)}^{\mu {\left(A\right)}^{-1}-1}\mu_{A}\left(T_{A}>j\right)\\ \; & =\sum_{j=\tau (A)}^{\mu {\left(A\right)}^{-1}-1}\mu \left(A;T_{A}>j\right)\\ \; & =\sum_{j=\tau (A)}^{\mu {\left(A\right)}^{-1}-1}\mu \left(T_{A}=j+1\right). \end{array}$$

In the last equality, we used Lemma 4. By (45), one obtains:

(46) $$\begin{array}{cc} \rho (A)\; & \geq \mu \left(T_{A}\leq \mu {\left(A\right)}^{-1}\right)-\mu \left(T_{A}\leq \tau \left(A\right)\right)\\ \; & =\mu (T_{A}\leq \mu {\left(A\right)}^{-1})-\tau \left(A\right)\mu \left(A\right) \end{array}$$

where the equality follows by stationarity and the definition of τ(A).

By Lemmas 1 and 2, we know that $\tau \left(A\right)\mu \left(A\right)\overset{n}{⟶}0$ uniformly. Thus, it is enough to find a strictly positive lower bound for $\mu (T_{A}\leq \mu {\left(A\right)}^{-1})$. Let:

$$N=\sum_{j=1}^{\mu {\left(A\right)}^{-1}}{𝟙}_{A}\circ \sigma^{j}\;$$

which counts the number of occurrences of *A* up to $\mu {\left(A\right)}^{-1}$. By the so-called second moment method,

(47) $$\mu \left(T_{A}\leq \mu {\left(A\right)}^{-1}\right)=\mu \left(N\geq 1\right)\geq \frac{E{\left(N\right)}^{2}}{E(N^{2})}.$$

Stationarity gives E(N)=1. It remains to prove that $E(N^{2})$ is bounded above by a constant. Expanding $N^{2}$, using stationarity and E(N)=1, we obtain:

(48) $$E\left(N^{2}\right)=1+2\sum_{j=1}^{\mu {\left(A\right)}^{-1}}\left(\mu {\left(A\right)}^{-1}-j\right)\;\mu \left(A\cap \sigma^{-j}\left(A\right)\right)\;.$$

Let us first consider the ϕ-mixing case. For j≥n, mixing gives $\mu \left(A\cap \sigma^{-j}\left(A\right)\right)\leq \mu {\left(A\right)}^{2}+\mu \left(A\right)\phi \left(j-n+1\right)$. Thus,

(49) $$\begin{array}{c} \sum_{j=n}^{\mu {\left(A\right)}^{-1}}\left(\mu {\left(A\right)}^{-1}-j\right)\;\mu \left(A\cap \sigma^{-j}\left(A\right)\right)\leq \frac{1}{2}+\sum_{\ell =0}^{\mu {\left(A\right)}^{-1}-n}\phi \left(\ell +1\right) \end{array}$$

where we used $\mu {\left(A\right)}^{-1}-j\leq \mu {\left(A\right)}^{-1}$ to get the last term.

For 1≤j≤n−1, as before $A^{\left(j\right)}\subset A^{\left(\lceil j/2\rceil \right)}$; thus:

$$\begin{array}{cc} \mu \left(A\cap \sigma^{-j}\left(A\right)\right)\; & =\mu \left(A\cap \sigma^{-n}\left(A^{\left(j\right)}\right)\right)\\ \; & \leq \mu \left(A\cap \sigma^{-n-\lfloor j/2\rfloor }\left(A^{\left(\lceil j/2\rceil \right)}\right)\right)\\ \; & \leq \mu \left(A\right)\left(\mu \left(A^{\left(\lceil j/2\rceil \right)}\right)+\phi \left(\left\lfloor j/2+1\right\rfloor \right)\right)\\ \; & \leq \mu \left(A\right)\left(Ce^{-c\lceil j/2\rceil }+\phi \left(\left\lfloor j/2+1\right\rfloor \right)\right)\;. \end{array}$$

Therefore,

(50) $$\begin{array}{c} \sum_{j=1}^{n-1}\left(\mu {\left(A\right)}^{-1}-j\right)\;\mu \left(A\cap \sigma^{-j}\left(A\right)\right)\leq \sum_{j=1}^{n-1}\left(Ce^{-c\lceil j/2\rceil }+\phi \left(\left\lfloor j/2+1\right\rfloor \right)\right). \end{array}$$

Therefore, by (49) and (50), the summability of ϕ concludes the proof for the ϕ-mixing case.

If μ is ψ-mixing, we separate the sum in (48) into three parts. First, recall the definition of $g_{0}$ in Section 3.2. For $1\leq j\leq g_{0}$, we bound the sum as follows:

$$\sum_{j=1}^{g_{0}}\left(\mu {\left(A\right)}^{-1}-j\right)\;\mu \left(A\cap \sigma^{-j}\left(A\right)\right)\leq \sum_{j=1}^{g_{0}}\mu {\left(A\right)}^{-1}\mu \left(A\right)=g_{0}.$$

For $g_{0}+1\leq j\leq g_{0}+n-1$, we have by ψ-mixing:

$$\left(\mu {\left(A\right)}^{-1}-j\right)\;\mu \left(A\cap \sigma^{-j}\left(A\right)\right)\leq \mu {\left(A\right)}^{-1}\mu \left(A\cap \sigma^{-n-g_{0}}\left(A^{\left(j-g_{0}\right)}\right)\right)\leq M\;\mu {\left(A\right)}^{-1}\mu \left(A\right)\mu \left(A^{\left(\ell \right)}\right)$$

where we denoted $\ell =j-g_{0}$. Thus:

$$\sum_{j=g_{0}+1}^{g_{0}+n-1}\left(\mu {\left(A\right)}^{-1}-j\right)\;\mu \left(A\cap \sigma^{-j}\left(A\right)\right)\leq M\sum_{\ell =1}^{n-1}Ce^{-c\ell }.$$

Finally, applying ψ-mixing again,

$$\sum_{j=g_{0}+n}^{\mu {\left(A\right)}^{-1}}\left(\mu {\left(A\right)}^{-1}-j\right)\;\mu \left(A\cap \sigma^{-j}\left(A\right)\right)\leq M\sum_{j=n+g_{0}}^{\mu {\left(A\right)}^{-1}}\mu {\left(A\right)}^{-1}\;\mu {\left(A\right)}^{2}\leq M,$$

concluding the proof of the ψ-mixing case. □

## Data Availability

Not applicable.
